# Supplementary figures and images for: Foveal processing of emotion-informative facial features
Source: PLoS One. 2021 Dec 2;16(12):e0260814. doi: 10.1371/journal.pone.0260814 (PMC8638924; doi:10.1371/journal.pone.0260814)

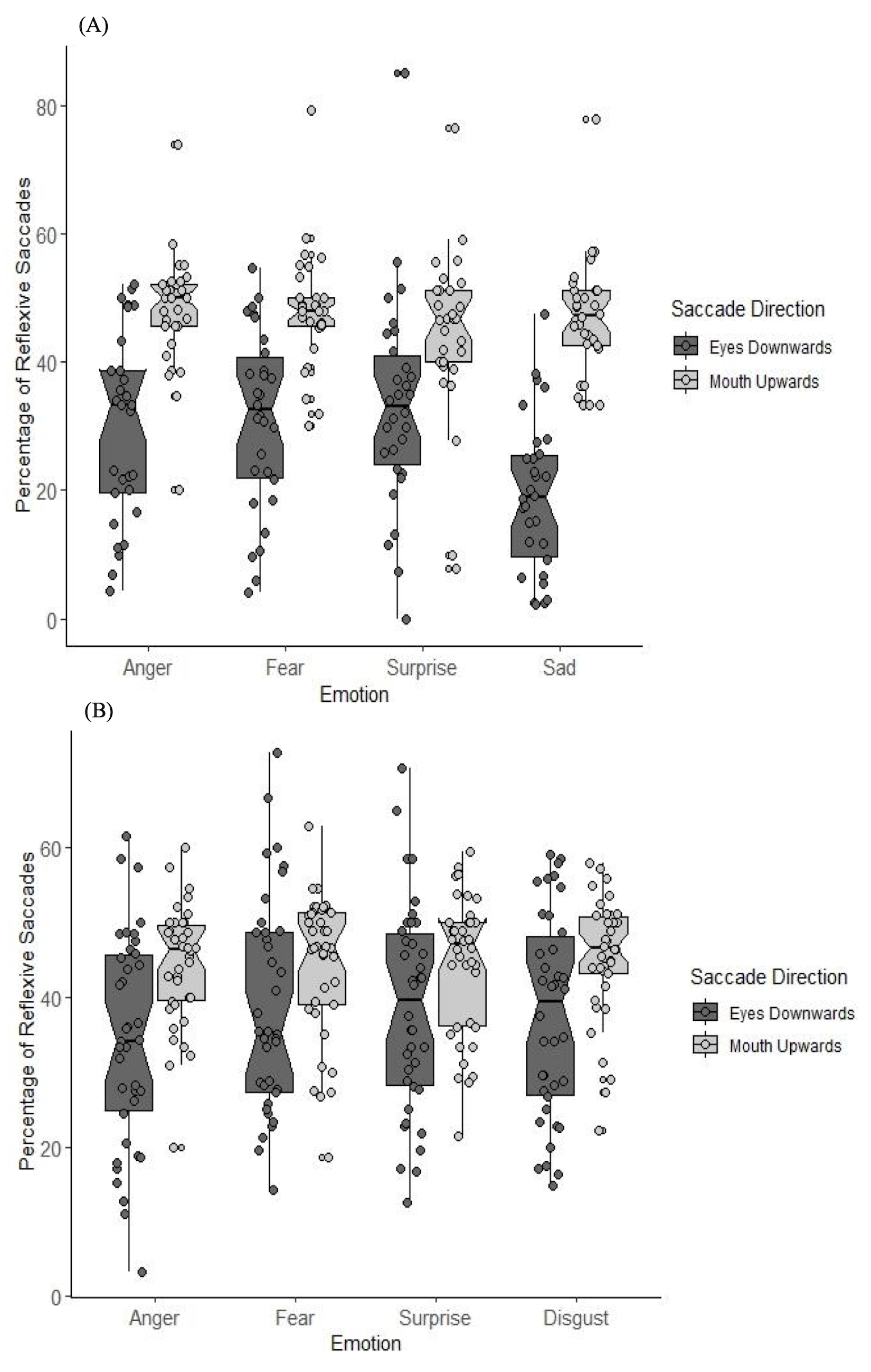

Supplement: S1 Fig — We compared the percentage of first saccades that were directed upwards from the mouth to the percentage of first saccades downwards from the eyes. Percentages for each emotion were calculated relative to the total number of first saccades (up or down) from fixation on the eyes and mouth combined. The percentage of saccades going upwards from the mouth was significantly higher than the percentage of saccades going downwards from the eyes for both Experiment 1 (a) and Experiment 2a (b). The interaction between emotion and saccade direction indicated that the percentage of saccades going downwards from the eyes was lower for sad faces compared to surprised faces for Experiment 1. The main effect of emotion for percentage of saccades going downwards from the eyes indicated that there were fewer saccades leaving the eyes for angry expressions compared to fearful and surprised expressions. In the figure, the median percentage is represented by the middle horizontal line and the notch on each boxplot. The upper and lower horizontal lines of each box delineate the interquartile range (upper line represents the 75th percentile and lower line represents the 25th percentile). The percentages of reflexive saccades for each participant are overlaid on top of the boxplot to represent the distribution of the data and outliers. (TIF) [file pone.0260814.s001.tif]

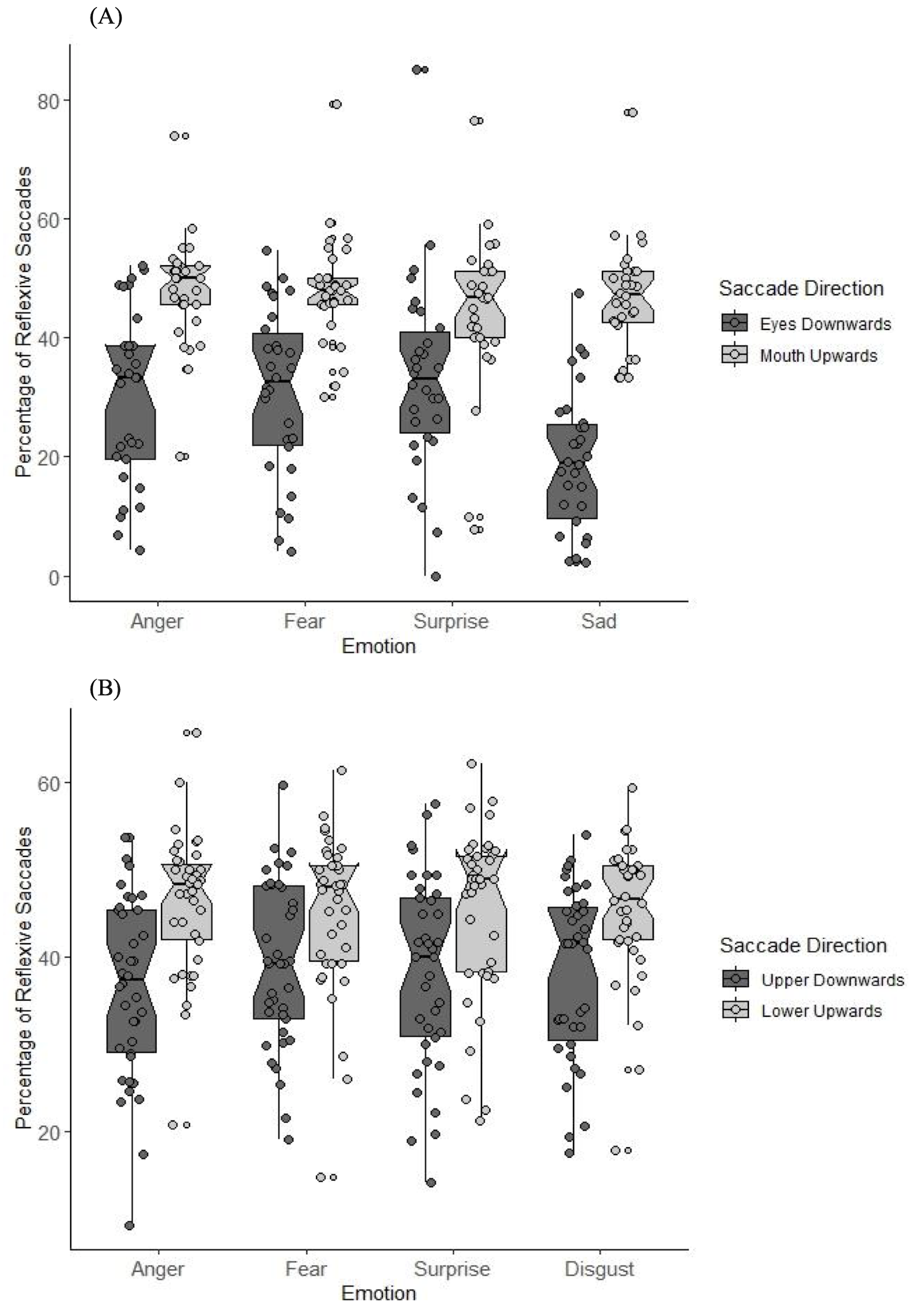

Supplement: S2 Fig — We compared the percentage of first saccades that were directed upwards from the lower facial features combined (cheeks + mouth) to the percentage of first saccades downwards from the upper facial features combined (eyes + brow). Percentages for each emotion were calculated as a percentage of the total number of initial saccades for each emotion per participant. The percentage of saccades going upwards from the lower features was significantly higher than the percentage of saccades going downwards from the upper features for both Experiment 1 (a) and Experiment 2a (b). Only in Experiment 2a, there were fewer saccades going downwards from upper features for angry faces compared to fearful faces. In the figure, the median percentage is represented by the middle horizontal line and the notch on each boxplot. The upper and lower horizontal lines of each box delineate the interquartile range (upper line represents the 75th percentile and lower line represents the 25th percentile). The percentages of reflexive saccades for each participant are overlaid on top of the boxplot to represent the distribution of the data and outliers. (TIF) [file pone.0260814.s002.tif]

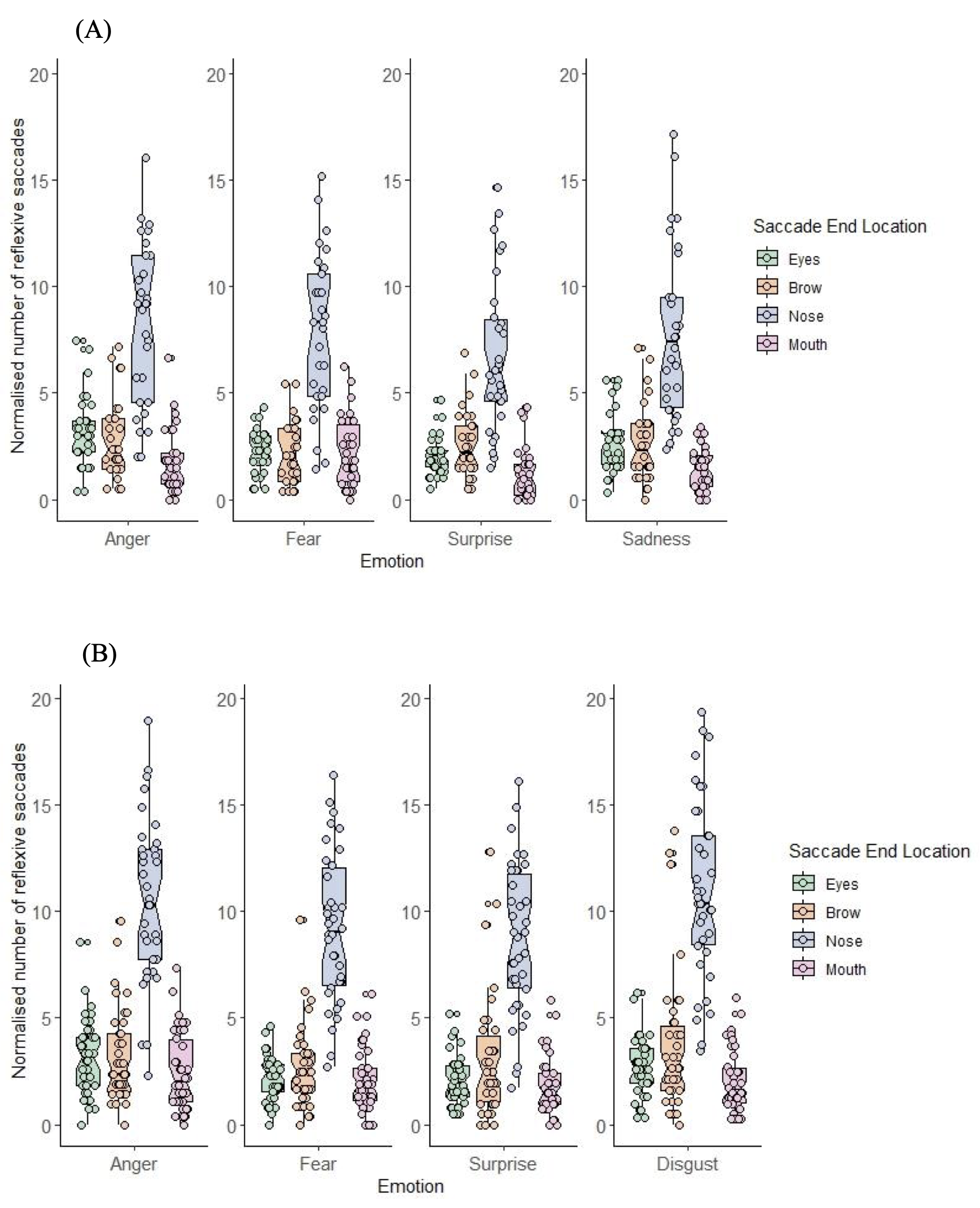

Supplement: S3 Fig — To investigate whether participants demonstrated a tendency to direct their fixations towards the centre of faces, we compared the mean frequency of first saccades ending in the eye, brow, nose and cheek regions of interest for each emotion. For the purposes of this analysis, we will accept the nose region as the centre of the face however it should be noted that the definition of the nose in this study comprises the area between the bridge and apex of the nose. We found that the first saccades ended in the nose region significantly more frequently than in the eyes, brow, or the mouth both in Experiments 1 (A) and 2a (B) indicating that the first saccades were somewhat affected by the centre-of-gravity effect. In the figure, the median percentage is represented by the middle horizontal line and the notch on each boxplot. The upper and lower horizontal lines of each box delineate the interquartile range (upper line represents the 75th percentile and lower line represents the 25th percentile). The percentages of reflexive saccades for each participant are overlaid on top of the boxplot to represent the distribution of the data and outliers. (TIF) [file pone.0260814.s003.tif]

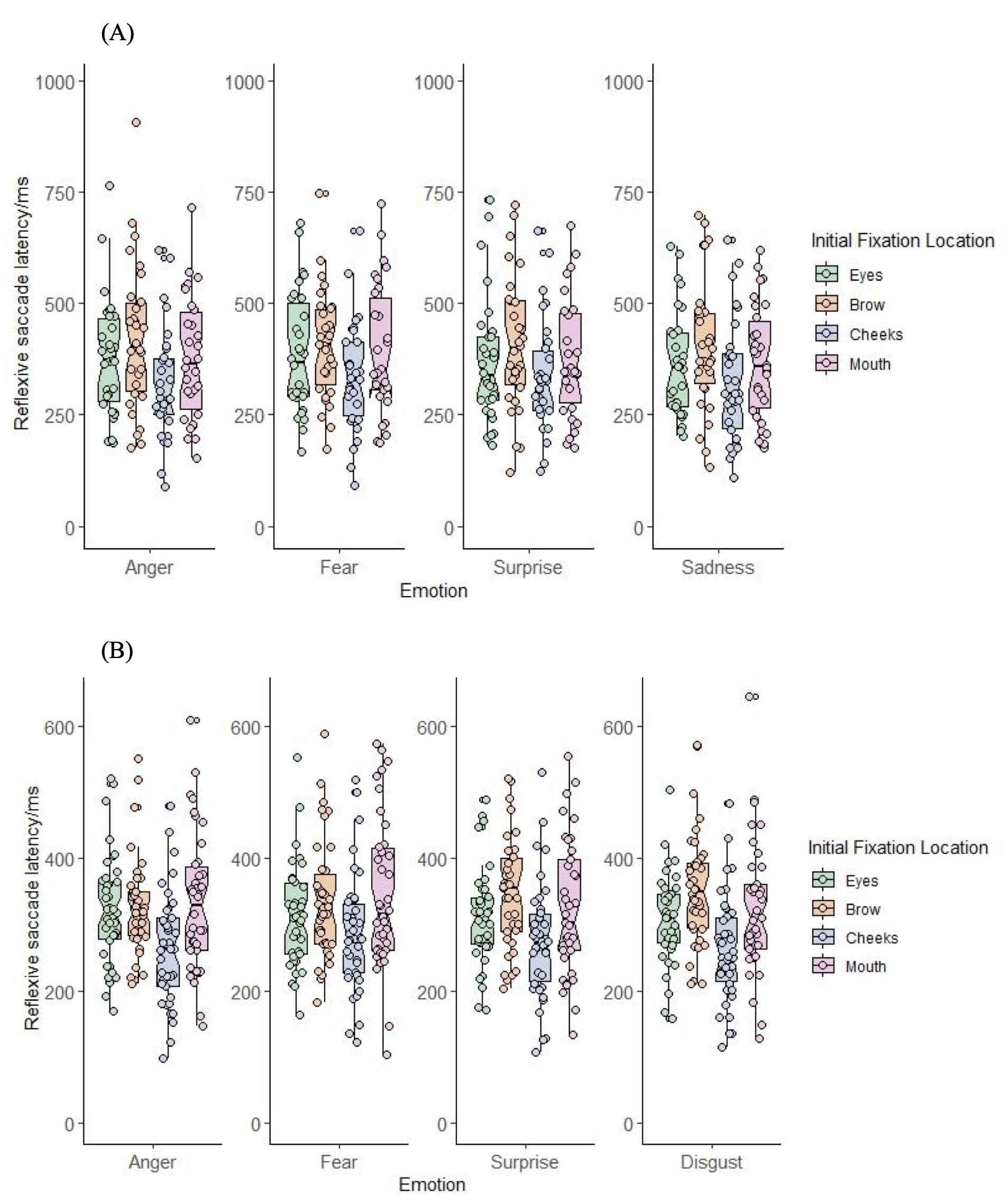

Supplement: S4 Fig — We investigated whether the initial fixation locations involved in this experiment influenced the latencies of reflexive first saccades. We found that the saccade latencies from initial fixation on the cheeks were shorter compared to all the other initial fixation locations for both Experiment 1 (A) and Experiment 2a (B). This indicates that the reflexive saccades in our study are influenced by the centre-of-gravity effect suggested by Bindemann et al. [32]. Only in Experiment 2a, the reflexive saccades from the eyes were shorter compared to reflexive saccades from the brow. Additionally, for Experiment 2a, we find that first saccade latencies from the brow for angry faces were shorter compared to disgusted faces and saccade latencies from the cheeks were longer for fear compared to anger and disgust faces. In the figure, the median percentage is represented by the middle horizontal line and the notch on each boxplot. The upper and lower horizontal lines of each box delineate the interquartile range (upper line represents the 75th percentile and lower line represents the 25th percentile). The percentages of reflexive saccades for each participant are overlaid on top of the boxplot to represent the distribution of the data and outliers. (TIF) [file pone.0260814.s004.tif]

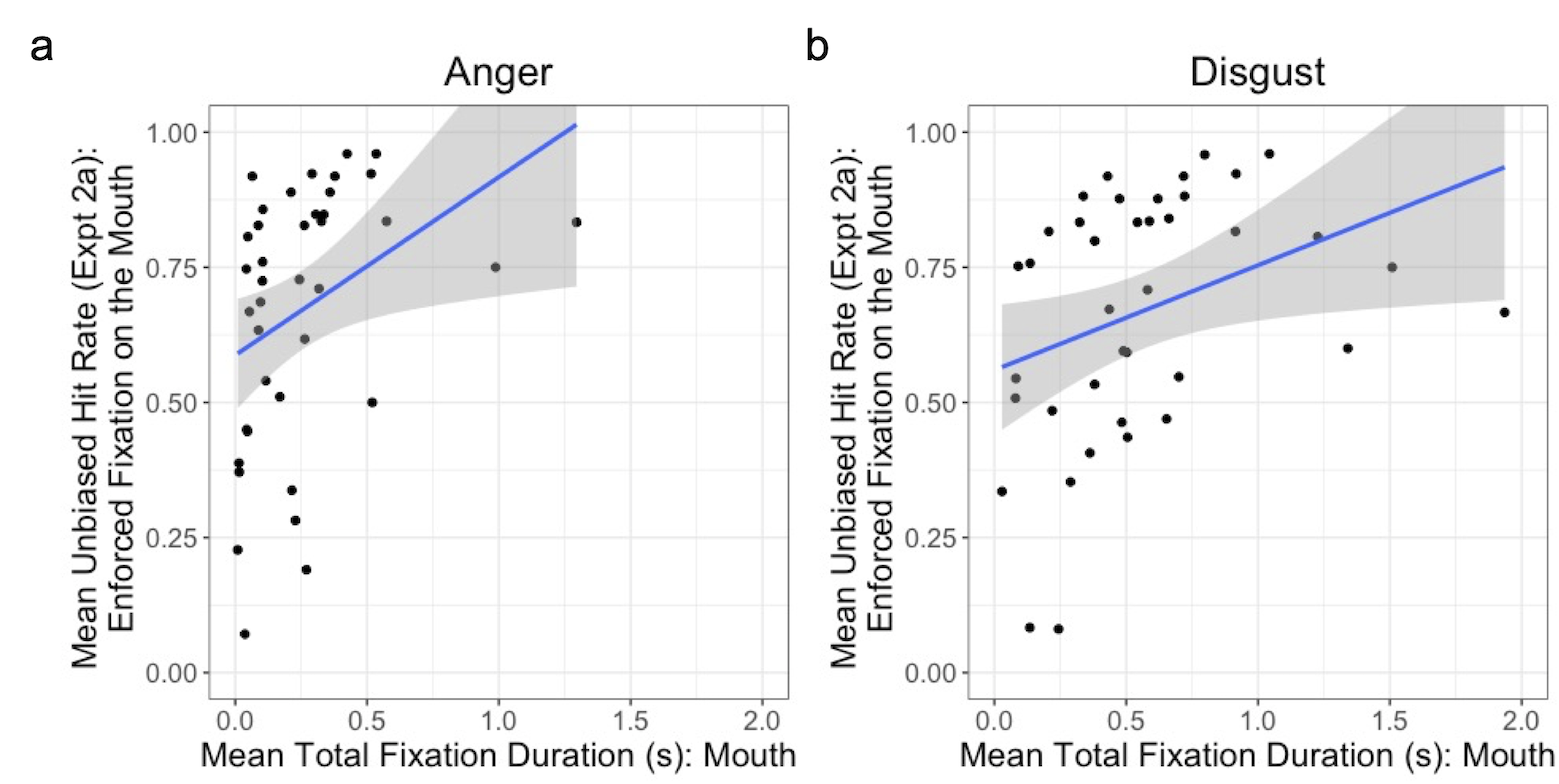

Supplement: S5 Fig — Panels show the associations between fixation duration on the mouth for (a) angry and (b) disgusted faces in Experiment 2b and emotion classification accuracy for those same emotions in Experiment 2a (brief fixation). Each dot represents a single participant. Shaded area indicates the 95% confidence interval. We found positive correlations between fixation duration on the mouth in Experiment 2b and emotion classification accuracy for angry and disgusted expressions when fixation was enforced on the mouth in Experiment 2a. (TIF) [file pone.0260814.s005.tif]

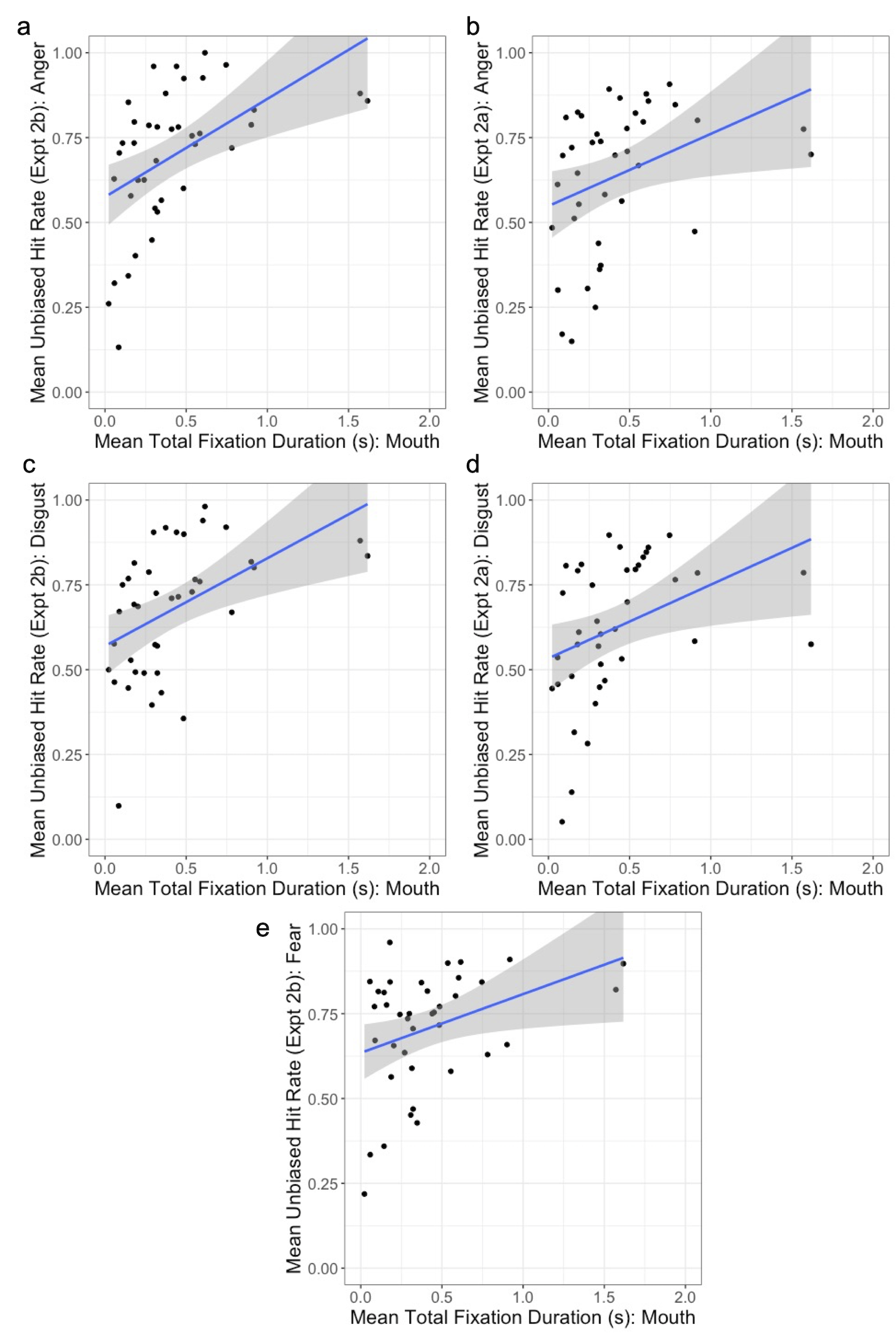

Supplement: S6 Fig — Panels show the associations between overall fixation duration on the mouth, regardless of emotion, in Experiment 2b and emotion classification accuracy for angry faces in (a) Experiment 2b (free viewing) and (b) Experiment 2a (brief fixation), and for disgusted faces in (c) Experiment 2b and (d) Experiment 2a, and for fearful faces in (e) Experiment 2b. Each dot represents a single participant. Shaded area indicates the 95% confidence interval. We found that fixating the mouth longer regardless of expression in Experiment 2b was positively correlated with anger and disgust classification accuracy in both Experiments 2a and 2b regardless of initial fixation location. Fixating the mouth longer in Experiment 2b was also marginally positively correlated with fear classification accuracy in Experiment 2b alone. (TIF) [file pone.0260814.s006.tif]
