# Supplementary material for: Foveal processing of emotion-informative facial features
Source: PLoS One. 2021 Dec 2;16(12):e0260814. doi: 10.1371/journal.pone.0260814 (PMC8638924; doi:10.1371/journal.pone.0260814)
Supplement: S1 Table — (PDF) [file pone.0260814.s007.pdf]

S1 Table. Results of pairwise comparisons for the saccade path analyses for Experiment 1: main effect of target location.

Target location contrast	*t*	*p*	d_z effect size [95% CI]
	From initial fixation on the left eye		
Brow > left cheek	7.57	< .001	1.456 [0.904 1.994]
Brow > mouth	6.69	< .001	1.288 [0.768 1.795]
Brow > right cheek	6.24	< .001	1.201 [0.696 1.692]
Brow > right eye	5.27	< .001	1.014 [0.541 1.473]
Right eye > left cheek	8.7	< .001	1.675 [1.079 2.257]
Right eye > mouth	7.97	< .001	1.534 [0.966 2.087]
Right eye > right cheek	7.73	< .001	1.487 [0.929 2.031]
Right cheek > left cheek	9.08	< .001	1.747 [1.136 2.345]
Right cheek > mouth	8.24	< .001	1.585 [1.007 2.149]
Mouth > left cheek	9.46	< .001	1.821 [1.194 2.435]
	From initial fixation on the right eye		
Brow > left cheek	10.0	< .001	1.924 [1.275 2.56]
Brow > mouth	10.27	< .001	1.976 [1.315 2.623]
Brow > right cheek	10.92	< .001	2.102 [1.413 2.777]
Brow > left eye	9.12	< .001	1.756 [1.143 2.355]
Left eye > left cheek	9.71	< .001	1.868 [1.231 2.355]
Left eye > mouth	9.91	< .001	1.907 [1.262 2.54]
Left eye > right cheek	10.76	< .001	2.07 [1.389 2.739]
Left cheek > mouth	9.47	< .001	1.823 [1.196 2.437]
Left cheek > right cheek	10.57	< .001	2.035 [1.361 2.695]
Mouth > right cheek	10.43	< .001	2.007 [1.34 2.662]
	From initial fixation on the right cheek		
Mouth > left cheek	3.67	.001	0.706 [0.278 1.123]
Mouth > left eye	3.56	.001	0.684 [0.259 1.099]
Mouth > brow	3.47	.002	0.667 [0.243 1.08]
Left cheek > left eye	3.26	.003	0.627 [0.208 1.035]

Left cheek > brow	3.12	.004	0.601 [0.185 1.007]
Mouth > right eye	3.12	.004	0.599 [0.184 1.005]
Left eye > brow	2.8	.009	0.539 [0.13 0.939]
Left cheek > right eye	2.58	.016	0.497 [0.092 0.893]

All df = 26, all p-values uncorrected. Only significant contrasts are shown. For each set of pairwise comparisons, minimum Bonferroni-Holm adjusted α = .005.
