# Supplementary material for: Foveal processing of emotion-informative facial features
Source: PLoS One. 2021 Dec 2;16(12):e0260814. doi: 10.1371/journal.pone.0260814 (PMC8638924; doi:10.1371/journal.pone.0260814)
Supplement: S2 Table — (PDF) [file pone.0260814.s008.pdf]

S2 Table. Results of pairwise comparisons for the saccade path analyses for Experiment 1: simple main effects for saccades from initial fixation on the right cheek.

Target location contrast	*t*	*p*	d_z effect size [95% CI]
	Angry faces		
Mouth > left cheek	3.19	.004	0.613 [0.196 1.02]
Mouth > left eye	3.06	.005	0.598 [0.175 0.994]
	Fearful faces		
Mouth > left eye	4.12	< .001	0.793 [0.353 1.221]
Mouth > left cheek	4.12	< .001	0.793 [0.353 1.221]
Mouth > brow	4.01	< .001	0.772 [0.335 1.197]
Mouth > right eye	3.71	< .001	0.714 [0.285 1.132]
Left cheek > left eye	3.92	< .001	0.755 [0.32 1.178]
Left cheek > brow	3.69	.001	0.71 [0.281 1.128]
Left cheek > right eye	3.21	.003	0.618 [0.201 1.026]
Left eye > brow	3.24	.003	0.623 [0.205 1.031]
Left eye > right eye	2.68	.012	0.517 [0.11 0.914]
Brow > right eye	2.33	.028	0.449 [0.049 0.842]
	Surprised faces		
Mouth > left cheek	3.55	.001	0.683 [0.258 1.098]
Mouth > left eye	3.51	.002	0.676 [0.252 1.09]
Mouth > brow	3.37	.002	0.649 [0.228 1.06]
Mouth > right eye	2.93	.007	0.564 [0.153 0.967]
Left cheek > left eye	3.25	.003	0.625 [0.207 1.033]
Left cheek > brow	2.95	.007	0.568 [0.156 0.97]

All df = 26. Only significant contrasts are shown. For each set of pairwise comparisons, minimum Bonferroni-Holm adjusted α = .005.
