# Supplementary material for: Foveal processing of emotion-informative facial features
Source: PLoS One. 2021 Dec 2;16(12):e0260814. doi: 10.1371/journal.pone.0260814 (PMC8638924; doi:10.1371/journal.pone.0260814)
Supplement: S3 Table — (PDF) [file pone.0260814.s009.pdf]

S3 Table. Results of pairwise comparisons for the saccade path analyses for Experiment 2a: main effect of target location.

Target location contrast	*t*	*p*	d_z effect size [95% CI]
From initial fixation on the left eye			
Right eye > left cheek	7.75	< .001	1.257 [0.825 1.679]
Right eye > mouth	5.27	< .001	0.855 [0.479 1.224]
Right eye > right cheek	4.2	< .001	0.682 [0.324 1.032]
Brow > left cheek	4.96	< .001	0.804 [0.433 1.166]
Brow > mouth	3.08	.004	0.5 [0.16 0.835]
Right cheek > left cheek	10.1	< .001	1.639 [1.145 2.124]
Right cheek > mouth	6.82	< .001	1.107 [0.697 1.508]
Mouth > left cheek	11.96	< .001	1.941 [1.393 2.479]
From initial fixation on the right eye			
Brow > left eye	5.08	< .001	0.823 [0.45 1.188]
Brow > mouth	5.0	< .001	0.81 [0.439 1.173]
Brow > left cheek	4.96	< .001	0.804 [0.433 1.167]
Brow > right cheek	5.28	< .001	0.856 [0.48 1.225]
Left eye > left cheek	4.63	< .001	0.751 [0.387 1.108]
Left eye > mouth	4.74	< .001	0.768 [0.402 1.127]
Left eye > right cheek	5.17	< .001	0.838 [0.463 1.205]
Left cheek > mouth	4.8	< .001	0.779 [0.411 1.139]
Left cheek > right cheek	5.36	< .001	0.87 [0.491 1.24]
Mouth > right cheek	5.55	< .001	0.9 [0.518 1.274]
From initial fixation on the left cheek			
Brow > left eye	3.9	< .001	0.633 [0.28 0.978]
Right eye > left eye	3.9	< .001	0.632 [0.28 0.977]
Right eye > brow	3.86	< .001	0.626 [0.274 0.971]
Right cheek > left eye	3.79	< .001	0.615 [0.264 0.959]
Right cheek > brow	3.69	< .001	0.598 [0.249 0.94]
Right cheek > right eye	3.56	.001	0.577 [0.23 0.918]

Mouth > left eye	3.19	.003	0.517 [0.175 0.853]
Mouth > brow	2.93	.006	0.476 [0.137 0.809]
Mouth > right cheek	2.15	.038	0.349 [0.02 0.675]
From initial fixation on the right cheek			
Mouth > left eye	4.44	< .001	0.72 [0.359 1.074]
Mouth > right eye	3.1	.004	0.503 [0.162 0.838]
Mouth > brow	3.92	< .001	0.636 [0.283 0.981]
Mouth > left cheek	5.41	< .001	0.878 [0.498 1.248]
Left cheek > left eye	2.85	.007	0.462 [0.124 0.794]

All df = 37, all p-values uncorrected. Only significant contrasts are shown. For each set of pairwise comparisons, minimum Bonferroni-Holm adjusted α = .005.
