# Supplementary material for: Foveal processing of emotion-informative facial features
Source: PLoS One. 2021 Dec 2;16(12):e0260814. doi: 10.1371/journal.pone.0260814 (PMC8638924; doi:10.1371/journal.pone.0260814)
Supplement: S4 Table — (PDF) [file pone.0260814.s010.pdf]

S4 Table. Results of pairwise comparisons for the fixation duration analyses of Experiment 2b: main effects of emotion.

Emotion contrast	*t*	*p*	d_z effect size [95% CI]
	Eyes		
Fear > anger	8.44	< .001	1.35 [0.91 1.78]
Fear > disgust	8.17	< .001	1.31 [0.88 1.73]
Fear > surprise	-1.2	.24	-0.19 [-0.51 0.13]
Surprise > anger	9.55	< .001	1.53 [1.06 1.99]
Surprise > disgust	8.02	< .001	1.28 [0.85 1.71]
Anger > disgust	0.62	.54	0.1 [-0.22 0.41]
	Brow		
Fear > anger	-6.24	< .001	-1.0 [-1.38 -0.61]
Fear > disgust	-8.58	< .001	-1.37 [-1.81 -0.93]
Fear > surprise	3.16	.003	0.51 [0.17 0.84]
Surprise > anger	-6.49	< .001	-1.04 [-1.43 -0.64]
Surprise > disgust	-8.31	< .001	-1.33 [-1.76 -0.89]
Anger > disgust	0.06	.95	0.01 [-0.3 0.32]
	Nose		
Fear > anger	-0.12	.91	-0.2 [-0.33 0.3]
Fear > disgust	-4.22	< .001	-0.68 [-1.02 -0.32]
Fear > surprise	0.53	.6	0.081 [-0.23 0.4]
Surprise > anger	-0.46	.65	-0.07 [-0.39 0.24]
Surprise > disgust	-4.36	< .001	-0.7 [-1.04 -0.34]
Anger > disgust	-4.55	< .001	-0.73 [-1.08 -0.37]
	Mouth		
Fear > anger	6.13	< .001	0.98 [0.59 1.36]
Fear > disgust	-5.86	< .001	-0.94 [-1.31 -0.56]
Fear > surprise	-1.9	.07	-0.3 [-0.62 0.02]
Surprise > anger	6.59	< .001	1.06 [0.66 1.44]
Surprise > disgust	-4.59	< .001	-0.74 [-1.09 -0.38]

Anger > disgust	-8.65	< .001	-1.39 [-1.82 -0.94]

All df = 38, all p-values uncorrected. For each set of pairwise comparisons, minimum Bonferroni-Holm adjusted α = .0083.
