# Supplementary material for: Foveal processing of emotion-informative facial features
Source: PLoS One. 2021 Dec 2;16(12):e0260814. doi: 10.1371/journal.pone.0260814 (PMC8638924; doi:10.1371/journal.pone.0260814)
Supplement: S5 Table — (PDF) [file pone.0260814.s011.pdf]

S5 Table. Results of pairwise comparisons for the fixation duration analyses of Experiment 2b: main effects of region of interest.

Fixation location contrast	*t*	*p*	d_z effect size [95% CI]
		Angry faces	
Eyes > brow	8.82	< .001	1.41 [0.96 1.85]
Eyes > nose	0.82	.42	0.13 [-0.19 0.45]
Eyes > mouth	7.51	< .001	1.2 [0.79 1.61]
Nose > brow	8.44	< .001	1.35 [0.91 1.78]
Nose > mouth	6.89	< .001	1.1 [0.7 1.5]
Mouth > brow	1.32	.2	0.21 [-0.11 0.53]
		Disgusted faces	
Eyes > brow	8.87	< .001	1.42 [0.97 1.86]
Eyes > nose	-0.05	.96	-0.01 [-0.32 0.31]
Eyes > mouth	3.8	< .001	0.61 [0.26 0.95]
Nose > brow	10.91	< .001	1.75 [1.24 2.25]
Nose > mouth	4.5	< .001	0.72 [0.36 1.07]
Mouth > brow	5.05	< .001	0.81 [0.44 1.17]
		Fearful faces	
Eyes > brow	12.0	< .001	1.92 [1.38 2.45]
Eyes > nose	2.3	.027	0.37 [0.04 0.69]
Eyes > mouth	6.96	< .001	1.11 [0.71 1.51]
Nose > brow	9.76	< .001	1.56 [1.09 2.03]
Nose > mouth	4.26	< .001	0.68 [0.33 1.03]
Mouth > brow	5.01	< .001	0.8 [0.44 1.16]
		Surprised faces	
Eyes > brow	13.07	< .001	2.09 [1.52 2.65]
Eyes > nose	2.7	.01	0.43 [0.1 0.76]
Eyes > mouth	6.99	< .001	1.12 [0.71 1.52]
Nose > brow	10.34	< .001	1.66 [1.17 2.14]
Nose > mouth	4.14	< .001	0.66 [0.31 1.01]

Mouth > brow	6.01	< .001	0.96 [0.58 1.34]

All df = 38, all p-values uncorrected. For each set of pairwise comparisons, minimum Bonferroni-Holm adjusted α = .0083.
