# Supplementary material for: Foveal processing of emotion-informative facial features
Source: PLoS One. 2021 Dec 2;16(12):e0260814. doi: 10.1371/journal.pone.0260814 (PMC8638924; doi:10.1371/journal.pone.0260814)
Supplement: S6 Table — (PDF) [file pone.0260814.s012.pdf]

S6 Table. Results of pairwise comparisons for the percentage fixation duration analyses of Experiment 2b: main effects of emotion.

Emotion contrast	*statisitc*	*p*	effect size [95% CI]
	Eyes		
Fear > anger	$t = 9.05$	< .001	$d_z = 1.45$ [0.99 1.9]
Fear > disgust	$t = 8.43$	< .001	$d_z = 1.35$ [0.91 1.78]
Fear > surprise	$t = -1.76$	.09	$d_z = -0.28$ [-0.6 0.04]
Surprise > anger	$t = 9.49$	< .001	$d_z = 1.52$ [1.05 1.98]
Surprise > disgust	$t = 8.65$	< .001	$d_z = 1.39$ [0.94 1.82]
Anger > disgust	$t = 1.12$	.27	$d_z = 0.18$ [-0.14 0.5]
	Brow		
Fear > anger	$W = 8$	< .001	$r_{rb} = -0.98$ [-0.99 -0.96]
Fear > disgust	$W = 3$	< .001	$r_{rb} = -0.99$ [-1.0 -0.98]
Fear > surprise	$W = 611$	.002	$r_{rb} = 0.57$ [0.28 0.76]
Surprise > anger	$W = 13$	< .001	$r_{rb} = -0.97$ [-0.98 -0.93]
Surprise > disgust	$W = 0$	< .001	$r_{rb} = -1.0$
Anger > disgust	$W = 364$	.73	$r_{rb} = -0.07$ [-0.4 0.29]
	Nose		
Fear > anger	$t = -0.35$	.73	$d_z = -0.06$ [-0.37 0.26]
Fear > disgust	$W = 170$	.002	$r_{rb} = -0.56$ [-0.76 -0.27]
Fear > surprise	$W = 414$	.75	$r_{rb} = 0.06$ [-0.29 0.4]
Surprise > anger	$t = -0.29$	.77	$d_z = -0.05$ [-0.36 0.27]
Surprise > disgust	$t = -3.8$	< .001	$d_z = -0.61$ [-0.95 -0.26]
Anger > disgust	$t = -4.5$	< .001	$d_z = -0.72$ [-1.07 -0.36]
	Mouth		
Fear > anger	$W = 753$	< .001	$r_{rb} = 0.93$ [0.86 0.97]
Fear > disgust	t = -5.37	< .001	$d_z = -0.86$ [-1.22 -0.49]
Fear > surprise	t = -1.86	.07	$d_z = -0.3$ [-0.62 0.03]
Surprise > anger	$W = 771$	< .001	$r_{rb} = 0.98$ [0.95 0.99]
Surprise > disgust	t = -4.09	< .001	$d_z = -0.66$ [-1.0 -0.31]
Anger > disgust	$W = 0$	< .001	$r_{rb} = -1.0$

All df = 38, all p-values uncorrected. For each set of pairwise comparisons, minimum Bonferroni-Holm adjusted $\alpha = .0083$.
