# Supplementary material for: Foveal processing of emotion-informative facial features
Source: PLoS One. 2021 Dec 2;16(12):e0260814. doi: 10.1371/journal.pone.0260814 (PMC8638924; doi:10.1371/journal.pone.0260814)
Supplement: S7 Table — (PDF) [file pone.0260814.s013.pdf]

S7 Table. Results of pairwise comparisons for the percentage fixation duration analyses of Experiment 2b: main effects of region of interest.

Fixation location contrast	*statistic*	*p*	effect size [95% CI]
	Angry faces		
Eyes > brow	$W = 780$	< .001	$r_{rb} = 1.0$
Eyes > nose	$t = 0.86$	.39	$d_z = 0.14$ [-0.18 0.45]
Eyes > mouth	$t = 7.92$	< .001	$d_z = 1.27$ [0.84 1.69]
Nose > brow	$W = 778$	< .001	$r_{rb} = 0.995$ [0.989 0.998]
Nose > mouth	$t = 7.17$	< .001	$d_z = 1.15$ [0.74 1.55]
Mouth > brow	$W = 458$	.35	$r_{rb} = 0.17$ [-0.18 0.49]
	Disgusted faces		
Eyes > brow	$t = 9.34$	< .001	$d_z = 1.5$ [1.03 1.95]
Eyes > nose	$t = -0.06$	.96	$d_z = -0.01$ [-0.32 0.31]
Eyes > mouth	$t = 3.96$	< .001	$d_z = 0.63$ [0.29 0.98]
Nose > brow	$t = 11.42$	< .001	$d_z = 1.83$ [1.31 2.34]
Nose > mouth	$t = 4.62$	< .001	$d_z = 0.74$ [0.38 1.09]
Mouth > brow	$t = 5.23$	< .001	$d_z = 0.84$ [0.47 1.2]
	Fearful faces		
Eyes > brow	$t = 12.58$	< .001	$d_z = 2.01$ [1.46 2.56]
Eyes > nose	$t = 2.35$	.024	$d_z = 0.38$ [0.05 0.7]
Eyes > mouth	$t = 7.3$	< .001	$d_z = 1.17$ [0.76 1.57]
Nose > brow	$W = 780$	< .001	$r_{rb} = 1.0$
Nose > mouth	$t = 4.43$	< .001	$d_z = 0.71$ [0.35 1.06]
Mouth > brow	$W = 743$	< .001	$r_{rb} = 0.91$ [0.81 0.95]
	Surprised faces		
Eyes > brow	$t = 13.52$	< .001	$d_z = 2.16$ [1.587 2.74]
Eyes > nose	$t = 2.83$	.007	$d_z = 0.45$ [0.12 0.78]
Eyes > mouth	$t = 7.31$	< .001	$d_z = 1.17$ [0.76 1.57]
Nose > brow	$W = 780$	< .001	$r_{rb} = 1.0$
Nose > mouth	$t = 4.28$	< .001	$d_z = 0.69$ [0.33 1.03]
Mouth > brow	$W = 775$	< .001	$r_{rb} = 0.99$ [0.97 1.0]

All df = 38, all p-values uncorrected. For each set of pairwise comparisons, minimum Bonferroni-Holm adjusted α = .0083.
