# Supplementary material for: Foveal processing of emotion-informative facial features
Source: PLoS One. 2021 Dec 2;16(12):e0260814. doi: 10.1371/journal.pone.0260814 (PMC8638924; doi:10.1371/journal.pone.0260814)
Supplement: S1 File — (PDF) [file pone.0260814.s014.pdf]

Supplementary Results and Discussion

for

Foveal processing of emotion-informative facial features

Nazire Duran, Anthony P. Atkinson

Experiment 1

Based on previous findings from studies using the brief-fixation paradigm [1–7], we expected to see a higher proportion of reflexive first saccades going upwards from fixation on the mouth compared to downwards from fixation on one of the eyes. Whether this effect would be modulated by the displayed emotion, as has been reported with some combinations of emotions [1–8] but not others [8], was considered an open question.

Supplementary Results: Saccade Analyses

We examined the percentages of reflexive first saccades upwards or downwards from the initial fixation location, to allow comparison with previous findings [1–7]. To this end, we performed two analyses. The first analysis provides a direct comparison with the findings of previous studies by comparing the percentage of first saccades that were directed upwards from the mouth with the percentage of first saccades downwards from the eyes. For this analysis, proportions for each emotion were calculated relative to the total number of first saccades (up or down) from fixation on the eyes and mouth combined. The second analysis compared the percentage of first saccades after stimulus offset that were directed upwards from initial fixation on one of the lower face locations (mouth or cheek) with the percentage of first saccades downwards from initial fixation on one of the upper face locations (brow or eye). For this second analysis, proportions for each emotion were calculated relative to the total number of first saccades (up or down) leaving the relevant fixation location. Repeated-

measures ANOVAs were used, with follow-up pairwise comparisons using t-tests or, where the data failed to meet the assumption of normality, Wilcoxon signed ranks tests.

Percentages of saccades up from mouth vs. down from eyes. A 4 × 2 repeated measures ANOVA was conducted using emotion and saccade direction as factors to compare the percentage of saccades going upwards from the mouth and downwards from the eyes for each emotion. Descriptive statistics can be seen in S1 Fig (panel A). There was a main effect of emotion, $F(1.47, 42.69) = 6.24$, $p = .008$, $\eta p^2 = .18$, and a main effect of saccade direction, $F(1, 29) = 39.28$, $p < .001$, $\eta p^2 = .58$. As expected, the percentage of saccades going upwards from the mouth was significantly higher than the percentage of saccades going downwards from the eyes. The Expression × Saccade Direction interaction was also significant, $F(2.16, 62.52) = 5.78$, $p = .004$. To further investigate this interaction, two separate one-way ANOVAs were carried out to investigate the effect of expression on the percentage of saccades going upwards from the mouth and downwards from the eyes separately.

There was a main effect of expression for saccades going downwards from the eyes, $F(1.63, 47.30) = 6.60$, $p = .005$, $\eta p^2 = .19$. The percentage of saccades going downwards from the eyes was lower for sad faces compared to surprised faces ($p < .05$). The main effect of expression did not reach significance for percentage of saccades going upwards from the mouth, $F(2.05, 51.59) = 2.57$, $p = .08$.

Four Wilcoxon signed-rank tests were carried out separately comparing percentage of saccades going downwards from the eyes and saccades going upwards from the mouth for each emotion. For all expressions, the Wilcoxon signed-rank tests showed that there was a significantly higher percentage of saccades going upwards from the mouth compared to saccades going downwards from the eyes (anger: $Z = -3.63$, $p < .001$; fear: $Z = -3.82$, $p < .001$; surprise: $Z = -2.53$, $p = .011$; sad: $Z = -4.68$, $p < .001$).

(A)

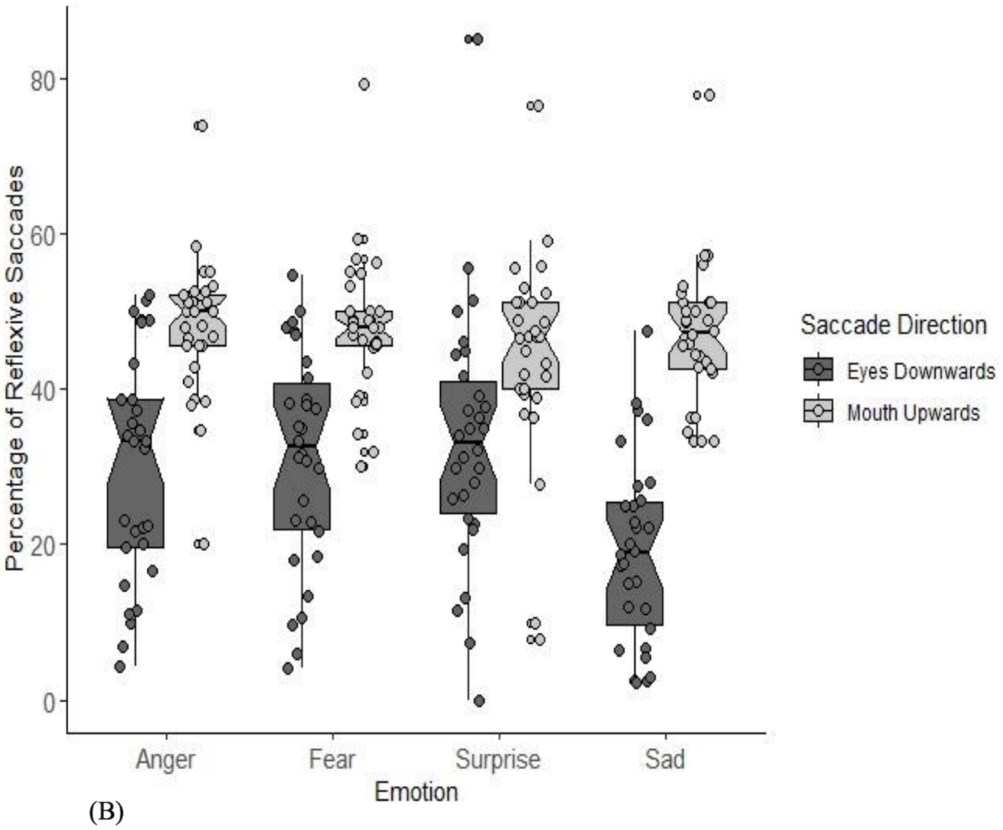

Percentage of Reflexive Saccades
0
20
40
60
80
Anger
Fear
Surprise
Sad
Emotion
Saccade Direction
Eyes Downwards
Mouth Upwards

(B)

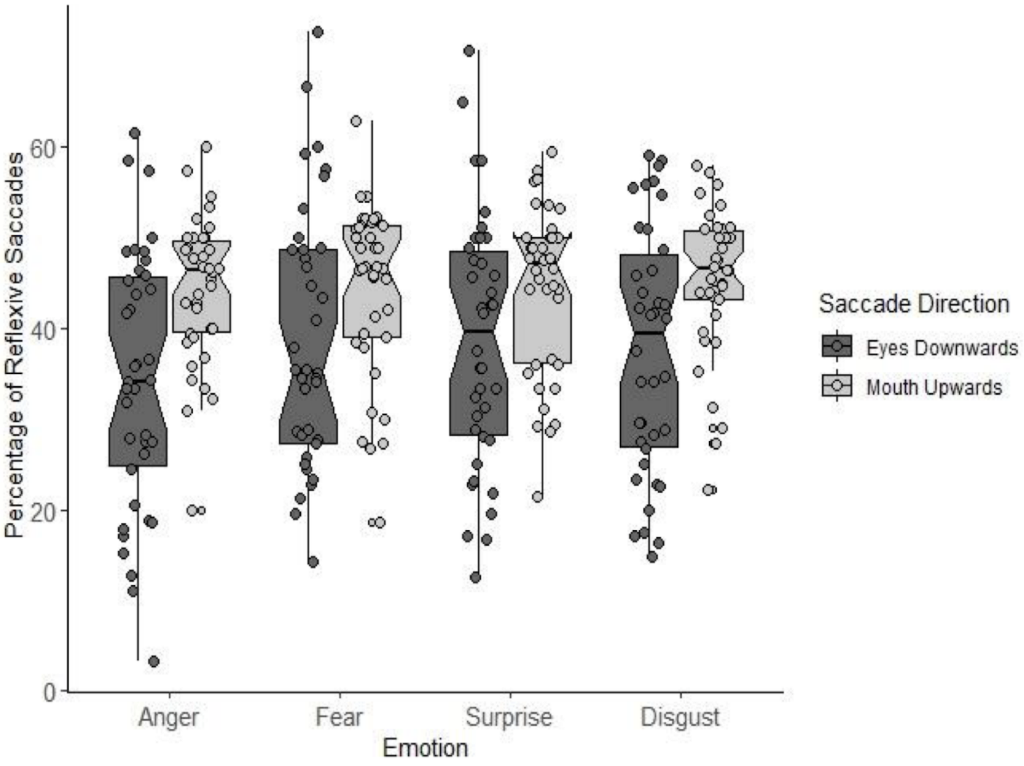

Percentage of Reflexive Saccades
0
20
40
60
Anger
Fear
Surprise
Disgust
Emotion
Saccade Direction
Eyes Downwards
Mouth Upwards

S1 Fig. The percentage of reflexive saccades going downwards from the eyes and upwards from the mouth for Experiment 1 and 2a.
We compared the percentage of first saccades that were directed upwards from the mouth to the percentage of first saccades downwards from the eyes. Percentages for each emotion were calculated relative to the total number of first saccades (up or down) from fixation on the eyes and mouth combined. The percentage of saccades going upwards from the mouth was significantly higher than the percentage of saccades going downwards from the eyes for both Experiment 1 (A) and Experiment 2a (B). The interaction between emotion and saccade direction indicated that the percentage of saccades going downwards from the eyes was lower for sad faces compared to surprised faces for Experiment 1. The main effect of emotion for percentage of saccades going downwards from the eyes indicated that there were fewer saccades leaving the eyes for angry expressions compared to fearful and surprised expressions. In the figure, the median percentage is represented by the middle horizontal line and the notch on each boxplot. The upper and lower horizontal lines of each box delineate the interquartile range (upper line represents the 75th percentile and lower line represents the 25th percentile). The percentages of reflexive saccades for each participant are overlaid on top of the boxplot to represent the distribution of the data and outliers.

Percentages of saccades up from lower features vs. down from upper features. Since we have included additional initial fixation locations to the original paradigm by Gamer & Büchel [4], we also calculated the percentage of saccades going downwards from upper initial fixation locations (i.e., the eyes and the brow) and upwards from lower initial fixation locations (i.e., the mouth and the cheeks) as a percentage of the total number of initial saccades for each emotion per participant. A 4 × 2 repeated measures ANOVA was conducted using emotion and fixation location as factors to compare these values. Descriptive statistics can be seen in S2 Fig (panel A). There was no main effect of emotion, $F(3, 87) = 1.11$, $p = .35$, $\eta p^2 = .037$. There was a main effect of fixation location, $F(1, 29) = 31.15$, $p < 0.01$, $\eta_p^2 = .52$. As expected, the percentage of saccades going upwards from lower features was significantly higher than the percentage of saccades going downwards from upper features. There was also a significant interaction, $F(3, 87) = 2.81$, $p = .04$, $\eta_p^2 = .088$.

There was no main effect of expression on the percentage of saccades going downwards from upper features, $F(3, 87) = 1.29$, $p = 0.28$, $\eta p^2 = .043$, but there was a significant effect of expression for saccades going upwards from lower facial features, $F(2.33, 67.43) = 3.72$, $p = 0.02$, $\eta_p^2 = .11$. Despite the effect of expression, however, there were no significant pairwise comparisons.

(A)

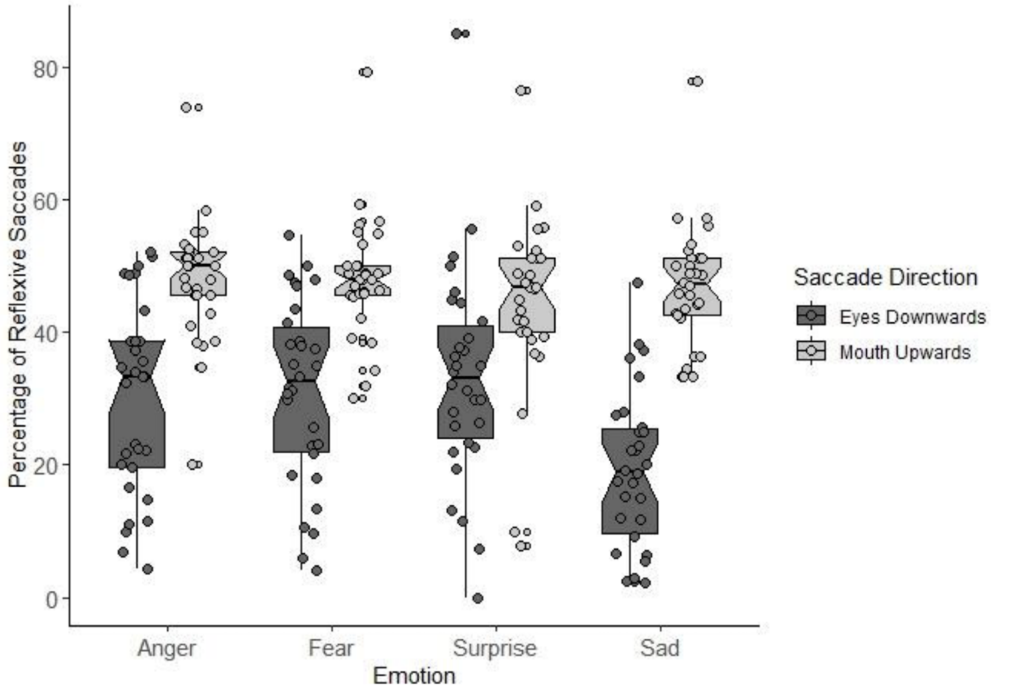

(B)

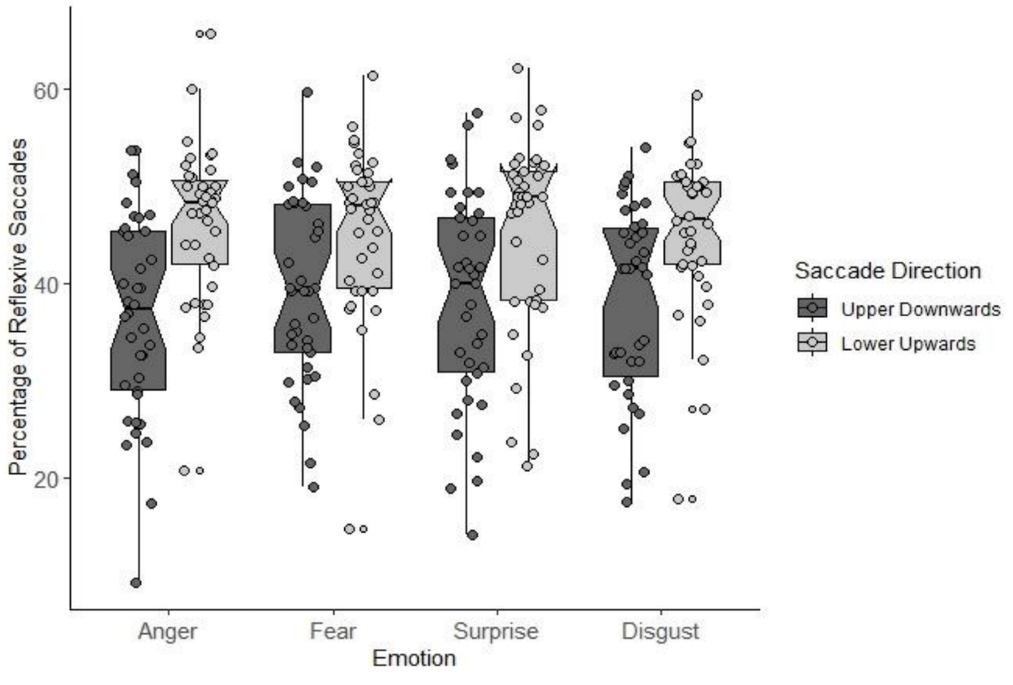

S2 Fig. The percentage of reflexive saccades going downwards from the upper features and upwards from the lower features for Experiments 1 and 2a.

We compared the percentage of first saccades that were directed upwards from the lower facial features combined (cheeks + mouth) to the percentage of first saccades downwards from the upper facial features combined (eyes + brow). Percentages for each emotion were calculated as a percentage of the total number of initial saccades for each emotion per participant. The percentage of saccades going upwards from the lower features was significantly higher than the percentage of saccades going downwards from the upper features for both Experiment 1 (a) and Experiment 2a (b). Only in Experiment 2a, there were fewer saccades going downwards from upper features for angry faces compared to fearful faces. In the figure, the median percentage is represented by the middle horizontal line and the notch on [illegible]

Wilcoxon signed-rank tests for angry (Z = -4.27, $p < .001$), fearful (Z = -4.55, $p <$ .001) and sad (Z = -4.10, $p < .001$) expressions revealed significantly higher percentage of saccades going upwards from the lower features compared to saccades going downwards from the upper features. A paired samples t-test for surprise showed that there was a higher percentage of saccades going upwards from the lower features compared to saccades going downwards from the upper features, $t_{(29)} = 4.05$, $p < .001$.

In summary, the percentage of reflexive first saccades going upwards from the mouth was higher than the percentage of reflexive first saccades going downwards from the eyes; similarly, the percentage of reflexive first saccades going upwards from the mouth and cheeks combined was higher than the percentage of reflexive first saccades going downwards from the eyes and brow combined. We did not, however, replicate previous findings that this effect is partially expression-specific [1–7], which argues against the idea that these saccades target expression-specific facial features. Nonetheless, we did find proportionately fewer reflexive first saccades were directed downwards from the eyes for briefly presented sad faces compared to surprised faces.

First saccade end location. We wanted to investigate whether our observers' initial saccades tended to target the central feature of the face (i.e., the nose). This analysis was motivated by Bindemann et al.'s [9] findings suggesting that early saccades are driven by the centre-of-gravity effect in face recognition and by findings in our previous study [8] suggesting a centre-of-gravity effect on reflexive first saccades in the same brief-fixation

paradigm used here. A 4 × 4 repeated measures ANOVA was conducted on the total number of saccades ending in the eyes, brow, nose and mouth regions of interest for each emotion (the same regions of interest delineated for Experiment 2b – see main paper). The number of saccades were area-normalized by dividing them by the percentage of the whole face area covered by the relevant region of interest (ROI). There was a main effect of emotion, $F(2.41, 69.88) = 37.96$, $p < .001$, $\eta p^2 = .57$, and a main effect of saccade end location, $F(1.50, 43.56) = 47.66$, $p < .001$, $\eta p^2 = .62$. The main effect of emotion indicated that significantly more first saccades were made for angry faces compared to all other expressions (all *p*s < .05) and fewer first saccades were made for fearful faces compared to angry and surprised faces (both *p*s < .001). The main effect of end location showed that significantly more first saccades ended in the nose region compared to any of the other regions of interest (all ps < .001). There was also an emotion × end location interaction, $F(5.88, 170.59) = 4.82$, $p < .001$, $\eta p^2 = .14$ (S3 Fig, panel A).

To investigate this interaction, planned comparisons were carried out to compare the number of saccades ending in informative facial features compared to relatively non-informative features. This resulted in three one-tailed, paired t-tests or Wilcoxon signed ranks tests (as appropriate) for each expression. The results were accepted as significant at multiple comparison corrected p-value of 0.017. For angry expressions, there were more saccades ending in the nose region compared to the brow region ($Z = -4.41$, $p < .001$). There was also a trend towards higher number of saccades ending in the brow region compared to the mouth region, however this comparison did not reach the corrected significance value ($Z = -2.34$, $p = .02$). For fearful expressions, comparing the saccades ending in the mouth region to other ROIs did not reveal any significant differences (mouth ~ eyes: $Z = -.30$, $p = .77$; mouth ~ brow: $Z = -0.15$, $p = .88$) except that there were significantly more saccades ending in the nose region compared to the mouth ($Z = -4.52$, $p < .001$). There were significantly fewer

saccades ending in the mouth region for surprised expressions compared to the eyes (Z = -3.43, p = .001), brow (Z = -2.93, p = .003) and the nose (Z = -4.78, p < .001).

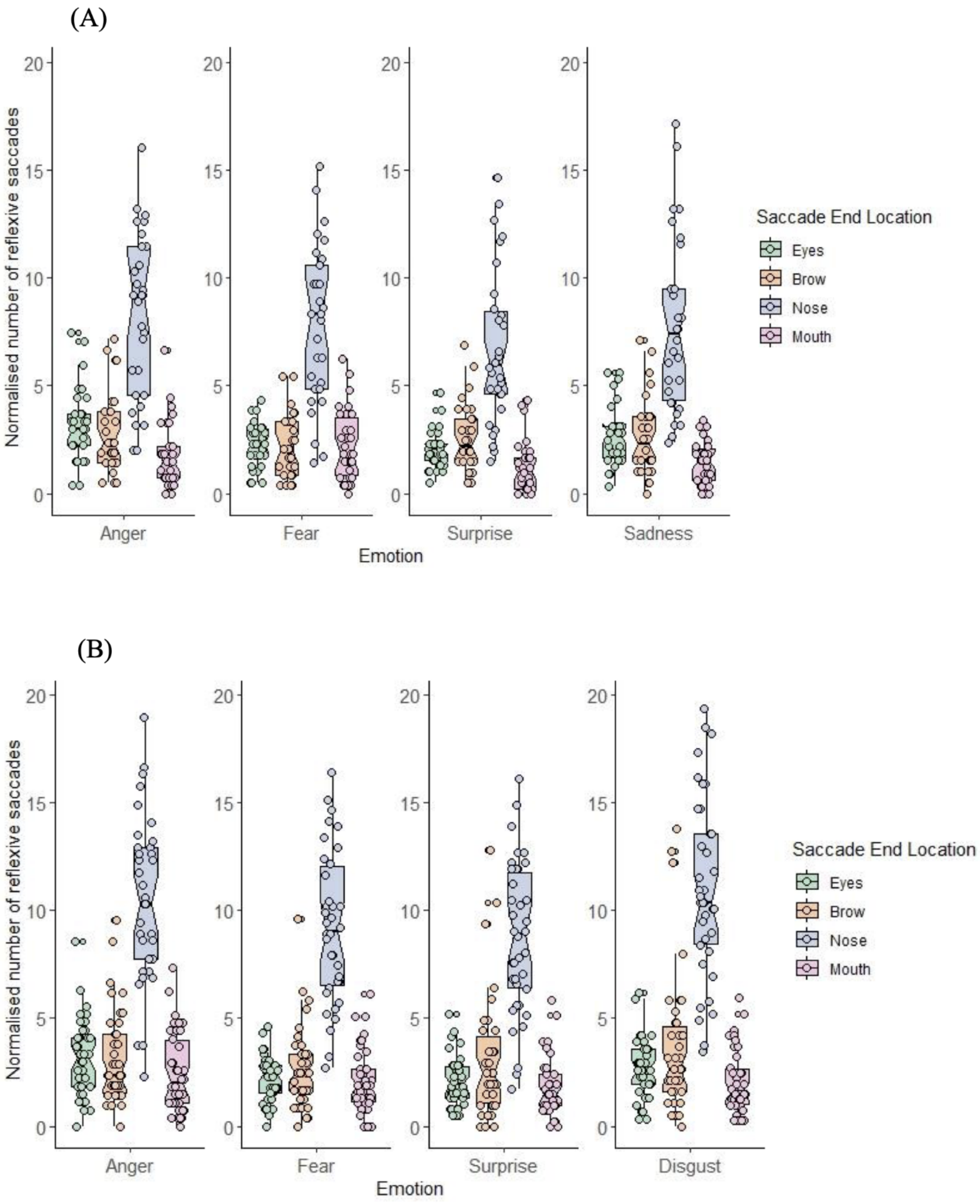

S3 Fig. Centre-of-gravity effect indexed by the mean frequency of first saccades ending in the eye, brow, nose and cheek ROIs for each emotion for Experiments 1 and 2a. To investigate whether participants demonstrated a tendency to direct their fixations towards the centre of faces, we compared the mean frequency of first saccades ending in the eye, brow, nose and cheek regions of interest for each emotion. For the purposes of this analysis, we will accept the nose region as the centre of the face however it should be noted that the definition of the nose in this study comprises the area between the bridge and apex of the nose. We found that the first saccades ended in the nose region significantly more frequently than in the eyes, brow, or the mouth both in Experiments 1 (A) and 2a (B) indicating that the

Latency of reflexive first saccades. Whether and when a saccade is generated is determined by two competing processes, one that promotes movement on the basis of visual information outside of foveal vision and one that maintains the eyes in their existing position, based at least in part on the visual information at the fixated location [e.g., 10]. We investigated whether the initial fixation locations involved in this experiment influenced the latencies of reflexive first saccades. If perceptual processing of the fixated region affects the temporal aspects of saccade generation, we expect saccades from the cheeks to have shorter latencies compared to the other initial fixation locations since the cheeks contain relatively less useful visual information for recognition accuracy. A 4 × 4 repeated measures ANOVA was conducted to compare the mean saccade latencies of the initial saccades for each expression at each initial fixation location. Descriptive statistics can be seen in S4 Fig (panel A). There was no main effect of emotion, $F(2.29, 66.51) = 1.58$, $p = .21$, and the Emotion × Fixation Location interaction was also not significant, $F(5.16, 149.58) = 0.59$, $p = .71$. However, there was a main effect of fixation location, $F(2.23, 66.64) = 8.21$, $p < .001$, $\eta_p^2 = .22$, indicating that the saccade latencies from initial fixation on the cheeks were shorter compared to all the other initial fixation locations as expected (all *p*s < .05).

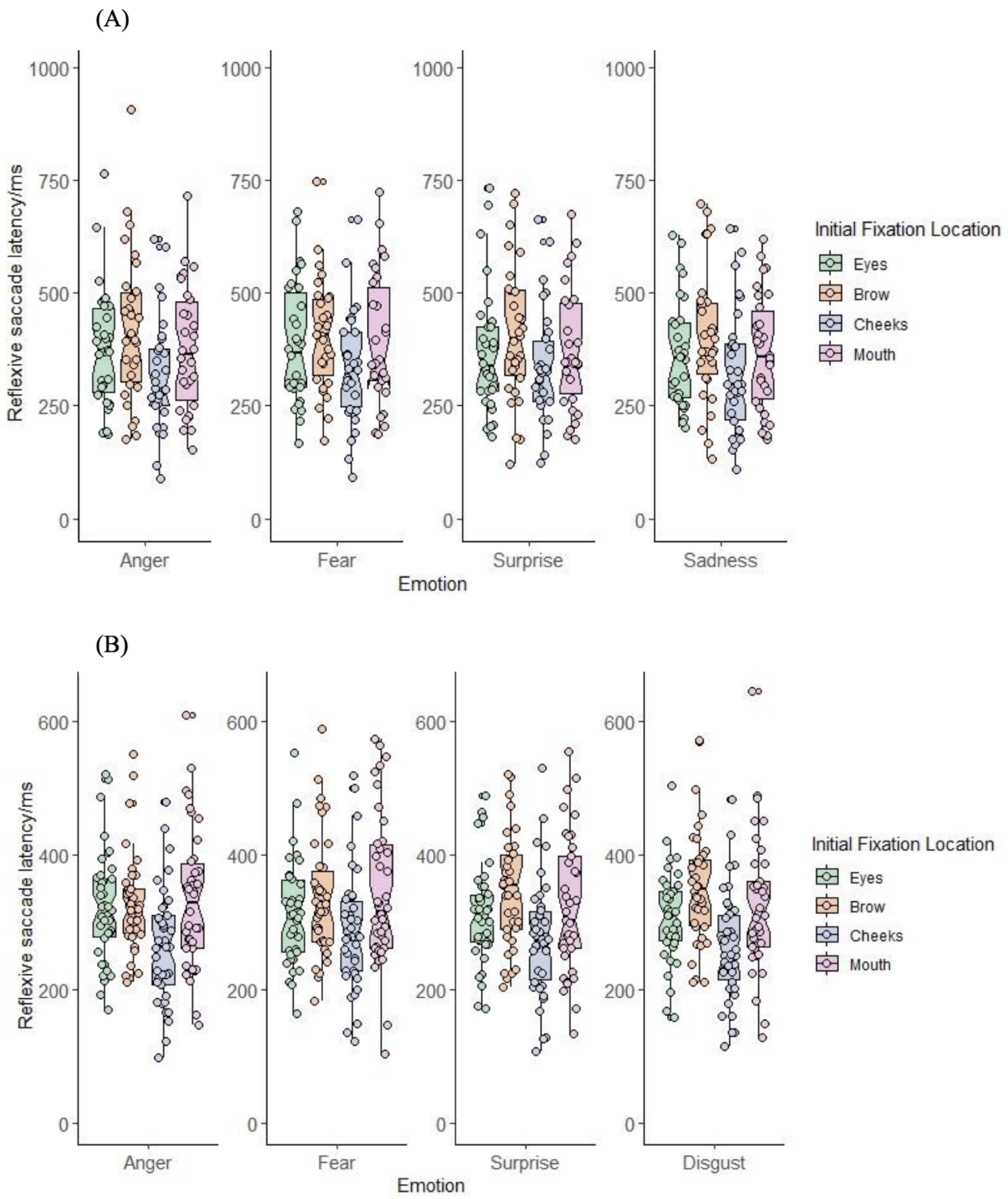

S4 Fig. The latency of reflexive saccades from each initial fixation location for Experiments 1 and 2a.

We investigated whether the initial fixation locations involved in this experiment influenced the latencies of reflexive first saccades. We found that the saccade latencies from initial fixation on the cheeks were shorter compared to all the other initial fixation locations for both Experiment 1 (a) and Experiment 2a (b). This indicates that the reflexive saccades in our study are influenced by the centre-of-gravity effect suggested by Bindemann et al. [9]. Only in Experiment 2a, the reflexive saccades from the eyes were shorter compared to reflexive saccades from the brow. Additionally, for Experiment 2a, we find that first saccade latencies from the brow for angry faces were shorter compared to disgusted faces and saccade latencies from the cheeks were longer for fear compared to anger and disgust faces. In the figure, the median percentage is represented by the middle horizontal line and the notch on each boxplot. The upper and lower horizontal lines of each box delineate the interquartile range (upper line represents the 75th percentile and lower line represents the 25th percentile). The percentages of reflexive saccades for each participant are overlaid on top of the boxplot to represent the distribution of the data and outliers.

within 1000ms from face offset, to be consistent with the analyses of first saccade data reported by other similar studies [1–7]. To minimize the number of voluntary or memory guided saccades and thus to limit the analyses to first saccades that are more likely to be 'reflexive', we also investigated the paths of first saccades that started 500ms after face offset. The valid saccade selection for this analysis is the same as reported in the main paper. A 4 × 6 repeated measures ANOVA (N=35) was used to compare the mean saccade paths towards each of the six possible saccade targets for each expression, similar to the saccade path analysis reported in the main paper. As opposed to the main analysis, while there was a main effect of target location, $F(5, 170) = 2.038$, $p = .076$, $\eta p^2 = .057$, this did not survive Greenhouse-Geisser correction (after GG correction: $F(2.14, 72.66) = 2.038$, $p = .135$, $\eta p^2 = .057$). There was no main effect of emotion, $F(3, 102) = 1.11$, $p = .35$, $\eta p^2 = .032$, nor an interaction, $F(7.84, 270.05) = 0.985$, $p = .469$, $\eta p^2 = .027$. This finding might indicate that initial saccades that can be more conservatively considered reflexive do not target informative facial features. It is possible that the memory of the recently presented face guides saccades towards the facial features that might be informative, as suggested by the significant interaction between emotion and target reported in the main paper.

Saccade path analysis: From fixation on individual features. From the left eye (N= 31), there was a main effect of target, $F(1.09, 32.67) = 18.40$, $p < .001$, $\eta p^2 = .38$, and the pairwise comparisons showed that, from the left eye, saccades targeted the right eye more strongly compared to all lower facial features (left cheek: $p < .001$; mouth: $p = .001$; right cheek: $p = .015$). The left cheek was the least strongly targeted feature (all *p*s < .05), followed by the mouth (left cheek and right cheek: *p*s < .001) and the right cheek. There was no main effect of emotion, $F(2.18, 65.47) = 0.54$, $p = .604$, $\eta p^2 = .018$ and the interaction between emotion and target did not survive Greenhouse-Geisser correction, $F(3.56, 106.82) = 1.90$, $p = .123$, $\eta p^2 = .060$.

From the brow (N=32), there was no main effect of target, $F(1.13, 35.05) = 1.67$, $p = .206$, $\eta p^2 = .051$, or an interaction between emotion and target, $F(3.78, 117.02) = 0.31$, $p = .863$, $\eta p^2 = .010$. The effect of emotion was also non-significant, $F(2.18, 67.51) = 2.24$, $p = .110$, $\eta p^2 = .067$.

From the right eye (N=32), the main effect of target, $F(1.06, 32.74) = 16.80$, $p < .001$, $\eta p^2 = .351$, and pairwise comparisons suggested that, from the right eye, saccades were more strongly targeting the brow compared to all other targets (left eye: $p = .001$, left cheek: $p = .002$, mouth: $p = .002$; right cheek: $p = .002$). Saccades were also more strongly targeting the left eye compared to all lower facial features (left cheek: $p = .006$, mouth: $p = .005$, right cheek: $p = .004$). Left cheek was targeted more strongly compared to mouth ($p = .007$) and the right cheek ($p = .007$) and the right cheek was the least strongly targeted facial feature (all *p*s < .005).

From the left cheek (N=32), the main effect of target, $F(1.06, 32.89) = 8.29$, $p = .006$, $\eta p^2 = .211$. This main effect suggested that saccades from the left cheek, more strongly targeted the right cheek compared to all upper features (left eye: $p = .013$; brow: $p = .017$; right eye: $p = .025$) and more strongly targeted the right eye compared to the left eye ($p = .010$) and the brow ($p = .009$) and more strongly targeted the brow compared to the left eye ($p = .011$).

From the mouth (N=32), there were no main effects of target, $F(1.14, 35.40) = 1.16$, $p = .297$, $\eta p^2 = .036$, emotion, $F(3, 93) = 0.159$, $p = .923$, $\eta p^2 = .005$, or an interaction, $F(3.21, 99.43) = 0.99$, $p = .462$, $\eta p^2 = .031$.

Finally, from the right cheek (N=32), there was a main effect of target, $F(1.04, 32.09) = 6.80$, $p = .013$, $\eta p^2 = .180$. The saccades from the right cheek more strongly targeted the mouth compared to the left eye ($p = .009$), brow ($p =.036$) and the left cheek ($p = .001$).

There was no main effect of emotion, $F(3, 93) = 0.48$, $p = .696$, $\eta p^2 = .015$, or an interaction, $F(3.07, 95.11) = 0.59$, $p = .627$, $\eta p^2 = .019$.

Supplementary Discussion

In line with previous findings [1–7], our results from the saccade direction analyses reported here in the Supplementary Results and Discussion show that observers' reflexive first saccades from initial fixation on the face more often went upwards from lower facial features than downwards from upper face features. However, unlike most of these previous studies, and consistent with the findings of Atkinson and Smithson [8], we did not find that this effect differed across emotional expressions. Nonetheless, there was a reduced tendency to shift gaze downwards from the eyes for sad faces, which might result from a reduced informativeness of the mouth region for this expression compared to angry, fearful and surprised expressions. Furthermore, the first saccades made by observers tended to end in the nose region regardless of the expression, which might be an indication of the centre-of-gravity effect [9]. Finally, latency of reflexive saccades from the cheeks was shortest confirming our expectation that there is less emotion-relevant information at the cheek region for any of the expressions. This, in turn, led to less visual processing at this region and faster saccades to more informative facial regions.

Experiment 2a

Supplementary Results: Saccade Analyses

Percentages of saccades up from mouth vs. down from eyes. To investigate whether there were proportionately more saccades made upwards from the mouth than downwards from the eyes and whether this varied as a function of the expressed emotion, we calculated the number of initial saccades going downwards from the eyes and upwards from the mouth as a proportion of the total number of saccades starting from the eyes and the mouth

combined. A 4 × 2 repeated measures ANOVA with emotion and initial fixation location as factors was conducted. The descriptive statistics can be seen in S1 Fig (panel B).

There was a main effect of emotion, $F(2.45, 90.64) = 5.61, p < .001, \eta p^2 = .13$, and a main effect of initial fixation, $F(1, 37) = 5.67, p = .01, \eta p^2 = .13$. The main effect of emotion indicated that there were fewer saccades for angry faces compared to fearful ($p < .001$), and disgusted faces ($p < .001$). The main effect of saccade direction indicated that there was a significantly higher percentage of saccades upwards from initial fixation on the mouth than downwards from initial fixation on the eyes ($p = .01$). There was no interaction between emotion and saccade direction, $F(3,111) = 2.02, p = .12$. Since Gamer and Büchel [4] found that the proportion of fixation changes from the mouth upwards was higher than the proportion of fixation going downward from the eyes and this effect was more pronounced for fearful (and neutral) faces, we wanted to further investigate whether the percentages of saccade direction was affected by facial expression used in this experiment as well. Two separate one-way ANOVAs were conducted for each saccade direction (eyes down, mouth up) comparing across emotions. There was a significant effect of emotion for saccades going downwards from the eyes, $F(3,111) = 4.19, p < .001, \eta p^2 = .10$. This main effect indicated that there were fewer saccades going downwards from the eyes for angry faces compared to fearful and surprised faces. There was no main effect of emotion for saccades going upwards from the mouth, $F(3,111) = 0.60, p = .62$.

A paired samples t-test for anger, $t(37) = 3.21, p = .003$, and a Wilcoxon signed-rank test for disgust, $Z = -2.24, p = .03$, showed that there was a higher percentage of saccades going upwards from the mouth compared to saccades going downwards from the eyes. Wilcoxon signed-rank tests for fear, $Z = -1.70, p = .09$, and surprised, $Z = -1.71, p = .09$, expressions revealed no significant difference between saccade directions.

Percentages of saccades up from lower features vs. down from upper features. Since we have used more initial fixation locations than the Gamer and Büchel [4] study and other studies using the brief-fixation paradigm, we further analysed the percentage of the reflexive first saccades downwards from the upper features (i.e., eyes and the brow) versus upwards from lower features (i.e., cheeks and the mouth) for each emotion, with the percentages calculated relative to the total number of reflexive first saccades for each emotion. A 4 × 2 repeated measures ANOVA with emotion and fixation location (lower face, upper face) as factors was conducted. The descriptive statistics can be seen in S2 Fig (panel B). There was no main effect of emotion, $F(2.54\ 94.05) = 0.87$, $p = .44$, $\eta p^2 = .023$. The main effect of fixation location, $F(1, 37) = 8.35$, $p = .006$, $\eta p^2 = .184$, indicated that there was a higher percentage of saccade going upwards from the lower features compared to downwards from upper features ($p < .001$). The interaction was not significant, $F(2.40, 88.96) = 2.24$, $p = .10$, $\eta p^2 =.057$.

Similar to the previous analysis, to further investigate whether expression had an effect on the direction of the first reflexive saccade, two separate one-way ANOVAs were conducted for each initial fixation location comparing across emotions. The main effect of emotion for saccades going downwards from the upper features was not significant, $F(1, 37) = 2.37$, $p = .07$, $\eta p^2 = .06$. Pairwise comparisons showed that there were fewer saccades going downwards from upper features for angry faces compared to fearful faces ($p = .02$). No main effect of emotion was present for saccades going upwards from lower features, $F(2.43, 90.14) = 1.02$, $p = .39$.

Wilcoxon signed-rank tests for anger, $Z = -3.19$, $p = .001$, fear, $Z = -2.28$, $p = .023$, surprise, $Z = -2.02$, $p = .043$, and disgust, $Z = -2.24$, $p = .03$, expressions showed that there was a higher percentage of saccades going upwards from the mouth compared to saccades going downwards from the eyes.

In summary, the percentage of reflexive first saccades going upwards from the mouth was higher than the percentage of reflexive first saccades going downwards from the eyes; similarly, the percentage of reflexive first saccades going upwards from the mouth and cheeks combined was higher than the percentage of reflexive first saccades going downwards from the eyes and brow combined. This replicates a finding from Experiment 1 and a basic finding from a number of other studies using the brief-fixation paradigm [1–7]. As with Experiment 1, however, we did not replicate the finding of most of those previous studies that this effect is partially expression-specific, which argues against the idea that these saccades target expression-specific facial features. Nonetheless, we did find that there were fewer reflexive first saccades directed downwards from the eyes and brow for angry faces compared to fearful and surprised faces.

First saccade end locations. To investigate whether our observers demonstrated a tendency to direct their fixations towards the centre of the briefly presented faces, we compared the mean frequency of first saccades ending in the eye, brow, nose and cheek regions of interest for each emotion. Since Bindemann et al. [9] showed that the early saccades in face recognition are directed to the geometric centre of the face stimuli, it is important to investigate whether the reflexive saccades in this experiment might also be affected by this centre-of-the gravity effect, as we did for Experiment 1. For the purposes of this analysis, we will accept the nose region as the centre of the face however it should be noted that the definition of the nose in this study comprises the area between the bridge and apex of the nose. Therefore, instead of the actual centre of the faces, it is more appropriate to indicate that this analysis will compare the mean frequency of first saccades that end within the central feature (i.e., the nose) of the presented faces. A 4 × 4 repeated measures ANOVA with emotion and region of interest as factors was conducted on the total number of reflexive saccades ending within each ROI (ROIs that were delineated as for Experiment 2b). The total

number of saccades was normalised by dividing them by the percentage of the whole face area covered by the relevant ROI. Graphical illustration of the descriptive statistics can be seen in S3 Fig (panel B).

There was a significant main effect of emotion, $F(3,111) = 55.69, p < .001, \eta p^2 = .60$, and a significant main effect of region of interest, $F(2.09, 77.43) = 85.95, p < .001, \eta p^2 = .70$. The main effect of emotion indicated that more saccades were made for angry and disgusted expressions compared to fearful and surprised expressions (all *p*s < .001). The main effect of region of interest reflected the fact that the first saccades ended in the nose region significantly more frequently than in the eyes, brow or the mouth ROIs (all *p*s < .001).

There was also a significant interaction between emotion and ROI, $F(5.42, 200.56) = 6.56, p < .001, \eta p^2 = .15$. Planned comparisons were carried out to further investigate this interaction. A Bonferroni-corrected p-value of 0.017 was used for multiple comparisons. For angry expressions, reflexive saccades did not end in the brow region significantly more compared to other initial fixation locations, and following the main effect of region of interest, more saccades ended in the nose region compared to the brow (brow ~ eyes: $Z = -0.20, p = .85$; brow ~ mouth: $Z = -0.88, p = .38$, brow ~ nose: $Z = -5.37, p < .001$). For fearful expressions, similarly, the first saccades tended to end significantly more in the nose region ($Z = -5.33, p < .001$) but no other comparison reached significance (mouth ~ eyes: Z = -0.62, $p = .54$; mouth ~ brow: $Z = 0.91, p = .37$). For surprised expressions, first saccades ended in the nose region, $Z = -5.31, p < .001$, and brow region, $Z = 2.37, p = .018$, significantly more compared to the mouth; there was no difference between the eyes and the mouth, $Z = -0.95, p = .34$. For disgusted expressions, saccades ended in the nose region significantly more compared to the mouth, $Z = -5.37, p < .001$.

Latency of reflexive first saccades. We next investigated the mean saccade latencies from each of the initial fixation locations for the reflexive first saccades, as we did for

Experiment 1. We expected the latencies from the cheeks to be shorter compared to the other fixation locations since we expect cheeks to have less diagnostic visual information compared to the eyes, brow and mouth. A 4 × 4 repeated measures ANOVA with emotion and initial fixation location as factors was carried out (S4 Fig, panel B).

There was no main effect of emotion, $F(3, 111) = 1.34, p = .27, \eta p^2 = .035$, but there was a significant main effect of initial fixation location, $F(1.80, 66.83) = 10.72, p < .001, \eta p^2 = .23$. This main effect indicated that the saccade latencies from the initial fixation location at one of the cheeks were significantly shorter than the saccade latencies from the eyes, the brow and the mouth (all ps < .001). Additionally, first saccade latencies were shorter from the eyes compared to the brow. There was also a significant interaction, $F(5.98, 221.42) = 2.68, p = .02, \eta p^2 = .07$.

To investigate this interaction, separate one-way ANOVAs were carried out for each fixation location separately. There was no main effect of emotion on saccade latencies for fixation at the eyes, $F(3,111) = 1.51, p = .22, \eta p^2 = .039$, or mouth, $F(3,111) = 0.71, p = .55, \eta p^2 = .019$. The significant main effect of emotion at the brow, $F(3,111) = 3.38, p = .02, \eta p^2 = .084$, indicated that first saccade latencies for angry faces were shorter compared to disgusted faces. Finally, the main effect of emotion for fixation at the cheeks, $F(3, 111) = 5.26, p < .002, \eta p^2 = .12$, indicated that first saccade latencies were longer for fear compared to anger and disgust.

To investigate whether the saccade latencies from the facial feature deemed informative for each expression was longer compared to relatively non-informative features, planned comparisons were carried out for each expression. This led to 3 one-tailed paired comparisons.

For angry facial expressions, comparing the saccade latencies from the brow to other initial fixation locations revealed that the latencies from the cheeks are significantly shorter

compared to latencies from the brow, $Z = -4.07$, $p < .001$. No other comparison was significant (brow ~ eyes: $Z = -0.56$, $p = .58$; brow ~ mouth: $Z = -0.30$, $p = .77$).

For fearful expressions, comparing latencies from the mouth to other initial fixation locations showed that saccade latencies from the mouth were shorter compared to the cheeks, $t(37) = 3.46$, $p = .001$. Despite a large numerical difference between latencies for saccades from the eyes and the mouth, this comparison did not reach significance, $t(37) = 1.79$, $p = .08$. There was no significant difference between the mouth and the brow, $t(37) = 0.49$, $p = .63$. For surprised facial expressions, latencies were significantly shorter for saccades from the cheeks compared to the mouth, $t(37) = 3.78$, $p = .001$; no other contrasts were significant.

For disgusted facial expressions, first saccade latencies from fixation on a cheek were significantly shorter than from fixation on the mouth ($Z = -3.98$, $p < .001$). Despite a large difference in latencies between the mouth and the brow fixation locations, implying that latencies from the brow were longer compared to the mouth, this did not reach significance ($Z = -1.68$, $p = .09$).

Supplementary Discussion

Similar to the findings of Experiment 1, observers in the brief-fixation paradigm (Experiment 2a) tended to make reflexive first saccades upwards from fixation on the lower facial features (mouth and cheeks) more than they did downwards from upper facial features (eyes and brow). Additionally, observers tended to make fewer reflexive first saccades downwards from the upper-face features for angry expressions compared to fearful and surprised expressions. We suggest that this reflects the relative informativeness of the mouth for fearful and surprised expressions compared to the brow for angry expressions. We also found some evidence to indicate that the first saccades were affected by the centre-of-gravity effect whereby most saccades ended in the nose region.

Experiment 2b

Relationship between total fixation duration and emotion recognition accuracy

As reported in the main paper, we found that time spent fixating the mouth was positively correlated with accuracy in classifying the emotions in Experiment 2b, particularly for disgust and anger. In a further, exploratory analysis reported here, we tested for relationships between time spent fixating emotion-informative facial regions in Experiment 2b and emotion recognition accuracy in the brief-fixation experiment (Experiment 2a), given that the same participants took part in both experiments. Anger classification accuracy with enforced fixation on the mouth in Experiment 2a was positively correlated with the amount of time spent fixating the mouth of angry faces in Experiment 2b, $\rho = .54$, $p < .001$ (S5 Fig, panel a). Yet anger classification accuracy with enforced fixation on the brow in Experiment 2a was not positively correlated with the amount of time spent fixating the brow of angry faces in Experiment 2b, $\rho = .2$, $p = .11$. Disgust classification accuracy with enforced fixation on the mouth in Experiment 2a was positively correlated with the amount of time spent fixating the mouth of disgusted faces in Experiment 2b, $\rho = .46$, $p = .002$ (S5 Fig, panel b). Yet disgust classification accuracy with enforced fixation on the brow in Experiment 2a was not positively correlated with the amount of time spent fixating the brow of disgusted faces in Experiment 2b ($p = .53$). Classification accuracy with enforced fixation on the mouth of either fearful or surprised faces in Experiment 2a was not significantly correlated with the amount of time spent fixating the mouth of, respectively, fearful or surprised faces in Experiment 2b (both *p*s > .27). Likewise, for both fearful and surprised faces, classification accuracy with enforced fixation on an eye in Experiment 2a was not positively correlated with the amount of time spent fixating the eyes of fearful and surprised faces, respectively, in Experiment 2b (both *p*s > .4).

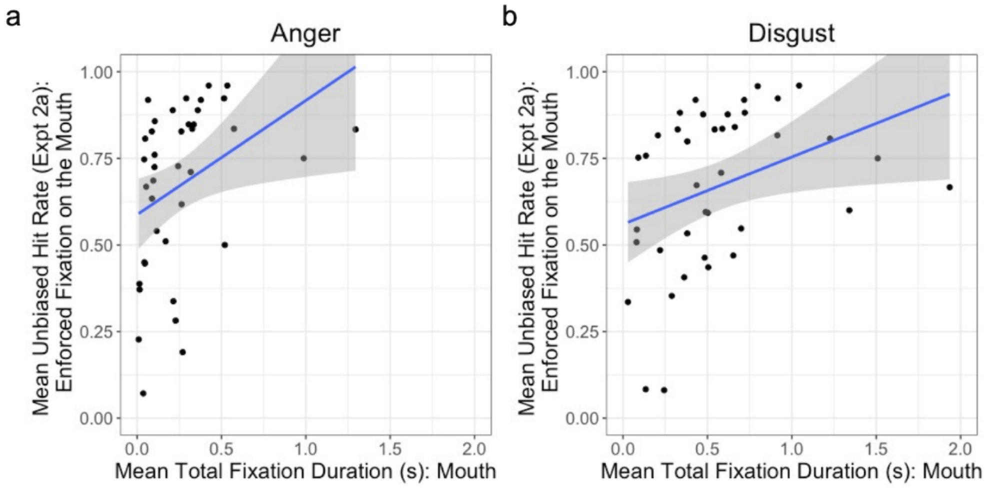

S5 Fig. Relationships between fixation duration on the mouth in Experiment 2b and emotion classification accuracy in Experiment 2a.
Panels show the associations between fixation duration on the mouth for (a) angry and (b) disgusted faces in Experiment 2b and emotion classification accuracy for those same emotions in Experiment 2a (brief fixation). Each dot represents a single participant. Shaded area indicates the 95% confidence interval. We found a positive correlation between emotion classification accuracy for angry and disgusted expressions when fixation was enforced on the mouth in Experiment 2a and fixation duration on the mouth in Experiment 2b.

Is fixation duration in any of the 4 ROIs (eyes, brow, nose, mouth), regardless of the emotion expressed on the face, related to accuracy in classifying any of the emotions? We conducted a further exploratory correlational analysis to address this question. Participants who spent longer fixating the mouth in Experiment 2b, irrespective of the emotional expression, were on average more accurate in classifying angry faces, not only in that same experiment, $\rho = .6$, $p < .001$ (S6 Fig, panel a), but also in the brief fixation paradigm of Experiment 2a (irrespective of the enforced fixation location), $\rho = .48$, $p = .002$ (S6 Fig, panel b). Similarly, participants who spent longer fixating the mouth in Experiment 2b were on average more accurate in classifying disgusted faces, not only in that same experiment, $\rho = .55$, $p < .001$ (S6 Fig, panel c), but also in the brief fixation paradigm of Experiment 2a, $\rho = .53$, $p < .001$ (S6 Fig, panel d). Time spent fixating the mouth was not correlated with accuracy for surprised expressions in either experiment (*p*s $\geq .09$) and was correlated with

accuracy for fearful expressions only marginally in Experiment 2b, $\rho = .32$, $p = .046$ (S6 Fig, panel e), and not at all in Experiment 2a ($p = .22$). Time spent fixating the eyes, nose or brow was not correlated with accuracy for any of the 4 emotions in either experiment (all ps > .075).

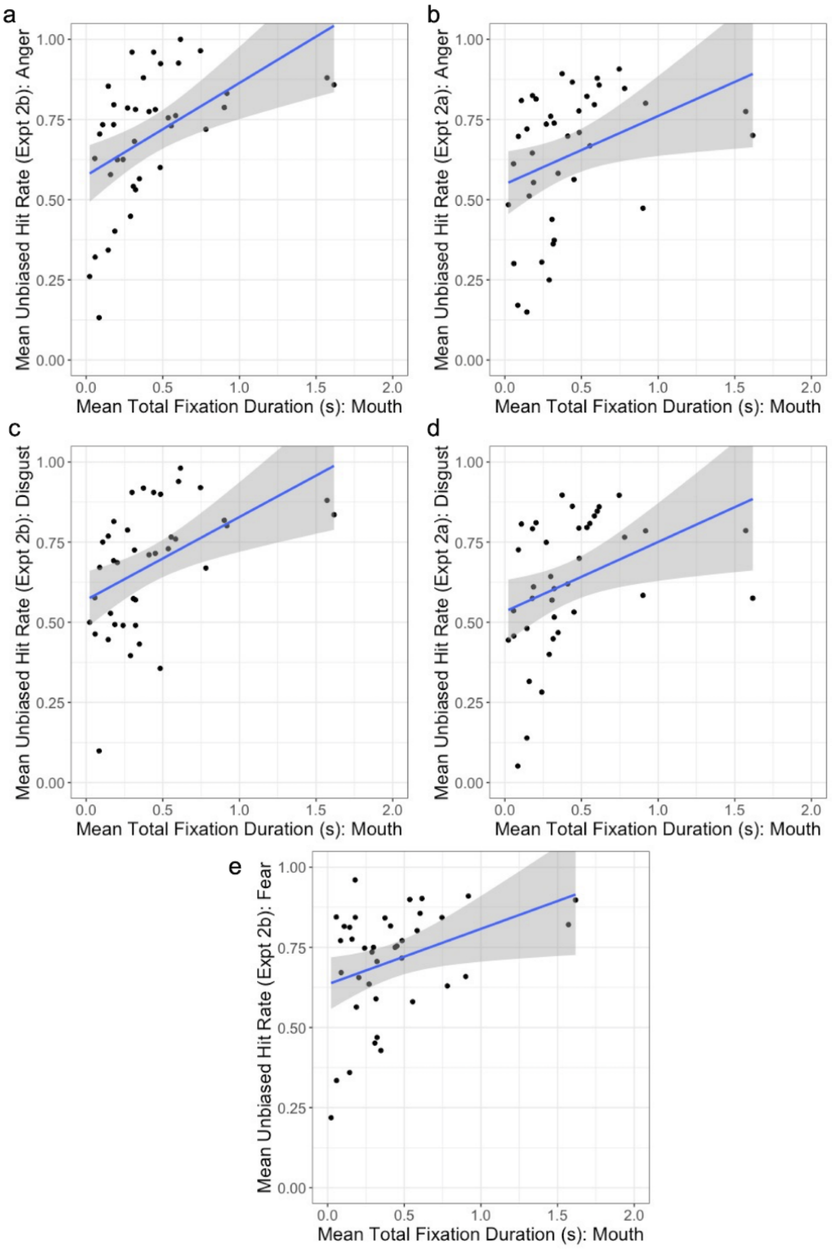

S6 Fig. Relationships between fixation duration on the mouth in Experiment 2b and emotion classification accuracy in Experiments 2a and 2b.
Panels show the associations between overall fixation duration on the mouth, regardless of emotion, in Experiment 2b and emotion classification accuracy for angry faces in (a) Experiment 2b (free viewing) and (b) Experiment 2a (brief fixation), and for disgusted faces in (c) Experiment 2b and (d) Experiment 2a, and for fearful faces in (e) Experiment 2b. Each dot represents a single participant. Shaded area indicates the 95% confidence interval. We found that fixating the mouth longer regardless of expression in Experiment 2b was positively correlated with anger and disgust classification accuracy in both Experiments 2a and 2b regardless of initial fixation location. Fixating the mouth longer in Experiment 2b was also marginally positively correlated with fear classification accuracy in Experiment 2b alone.

Mean percentage fixation duration

In the main paper, our eye movement analysis for Experiment 2b used as the dependent measure the total fixation duration on each ROI for each emotion. An alternative analysis strategy is to use the percentage or proportion of fixation times, relative to the total fixation duration on the image. This controls for any differences in image viewing times across conditions and participants. We therefore repeated our analyses of fixation durations on facial ROIs, this time to examine whether participants spent *proportionately* more time fixating the informative facial features for each of the expressions in the experiment. The results of this alternative analysis, reported below, were very similar to those reported in the main paper for the total fixation duration.

For each trial we calculated the total fixation duration, up to the point of the participant's button press, for the eyes, brow, nose and mouth ROIs and expressed these values as percentages relative to the total fixation duration on the image for that trial. Mean percentage fixation time values were then calculated for each ROI per emotion per participant. A repeated measures ANOVA on these percentage fixation times revealed significant main effects of emotion, $F(3, 114) = 86.07$, $p < .001$, $\eta p^2 = .694$, 90% CI [.61 .74], $\eta_G^2 = .008$, and region of interest, $F(1.61, 61.26) = 43.24$, $p < .001$, $\eta p^2 = .532$, 90% CI [.38 .63], $\eta_G^2 = .511$. The main effect of emotion reflected that participants spent proportionately less time fixating one or more of the 4 ROIs (eyes, brow, nose, mouth) for angry faces (M = 18.39%, SD = 2.35) than they did for fearful (M = 20.85%, SD = 1.94), surprised (M = 21.13%, SD = 1.67) and disgusted (M = 21.1%, SD = 1.73) faces (uncorrected ps < .001, d_zs

> 1.85; all other uncorrected *p*s > .2). The main effect of ROI showed that participants spent proportionately more time fixating the eyes (*M* = 36.36%, *SD* = 17.05) than the brow (*M* = 3.81%, *SD* = 2.63; uncorrected $p < .001$, $d_z = 1.863$) and mouth (*M* = 12.49%, *SD* = 9.97; uncorrected $p < .001$, $d_z = 1.088$) but not nose (*M* = 28.8%, *SD* = 14.61; uncorrected $p = .122$, $d_z = 0.253$), and proportionately more time fixating the nose than the brow (uncorrected $p < .001$, $r_{rb} = 1.0$) and mouth (uncorrected $p < .001$, $d_z = 0.83$), and longer for the mouth than for the brow (uncorrected $p < .001$, $r_{rb} = 0.782$).

There was also a significant Emotion × ROI interaction, $F(4.99, 189.53) = 35.96$, $p < .001$, $\eta p^2 = .486$, 90% CI [.39 .54], $\eta_G{}^2 = .051$. Simple main effects analyses revealed significant effects of ROI for each of the 4 emotions (*F*s ≥ 30.43, *p*s < .001) as well as significant effects of emotion for each of the 4 ROIs (*F*s ≥ 6.87, *p*s < .001). Pairwise comparisons for the main effects of emotion are reported in S6 Table, which reveal several findings consistent with our hypothesis that observers will spend more time fixating emotion-distinguishing than less informative facial features. Notably, participants spent proportionately more time fixating (1) the eyes for fearful and surprised faces than for angry and disgusted faces; (2) the brow for angry and disgusted faces than for fearful and surprised faces; (3) the nose for disgusted faces than for fearful, surprised and angry faces; and (4) the mouth for fearful and surprised faces than for angry faces and for disgusted faces than for angry, fearful and surprised faces. Additional pairwise comparisons comparing across ROIs for each emotion, reported in S7 Table, further revealed (5) that participants spent relatively more time fixating the mouth than the brow for disgusted, fearful and surprised faces, but for angry faces percentage dwell time did not differ between the brow and the mouth. Given the importance of the eyes in fearful and surprised faces and their visual similarity across these two emotions, it is also interesting to note that there were small tendencies for participants to spend more time fixating the eyes than the nose for fearful and surprised faces but not for

angry and disgusted faces, though after corrections for multiple comparisons, this effect was statistically significant only for surprised faces.

S6 Table. Results of pairwise comparisons for the percentage fixation duration analyses of Experiment 2b: main effects of emotion.

Emotion contrast	*statisitc*	*p*	effect size [95% CI]
	Eyes		
Fear > anger	$t = 9.05$	< .001	$d_z = 1.45$ [0.99 1.9]
Fear > disgust	$t = 8.43$	< .001	$d_z = 1.35$ [0.91 1.78]
Fear > surprise	$t = -1.76$	.09	$d_z = -0.28$ [-0.6 0.04]
Surprise > anger	$t = 9.49$	< .001	$d_z = 1.52$ [1.05 1.98]
Surprise > disgust	$t = 8.65$	< .001	$d_z = 1.39$ [0.94 1.82]
Anger > disgust	$t = 1.12$	.27	$d_z = 0.18$ [-0.14 0.5]
	Brow		
Fear > anger	$W = 8$	< .001	$r_{rb} = -0.98$ [-0.99 -0.96]
Fear > disgust	$W = 3$	< .001	$r_{rb} = -0.99$ [-1.0 -0.98]
Fear > surprise	$W = 611$	.002	$r_{rb} = 0.57$ [0.28 0.76]
Surprise > anger	$W = 13$	< .001	$r_{rb} = -0.97$ [-0.98 -0.93]
Surprise > disgust	$W = 0$	< .001	$r_{rb} = -1.0$
Anger > disgust	$W = 364$	.73	$r_{rb} = -0.07$ [-0.4 0.29]
	Nose		
Fear > anger	$t = -0.35$	.73	$d_z = -0.06$ [-0.37 0.26]
Fear > disgust	$W = 170$	.002	$r_{rb} = -0.56$ [-0.76 -0.27]
Fear > surprise	$W = 414$	.75	$r_{rb} = 0.06$ [-0.29 0.4]
Surprise > anger	$t = -0.29$	.77	$d_z = -0.05$ [-0.36 0.27]
Surprise > disgust	$t = -3.8$	< .001	$d_z = -0.61$ [-0.95 -0.26]
Anger > disgust	$t = -4.5$	< .001	$d_z = -0.72$ [-1.07 -0.36]
	Mouth		
Fear > anger	$W = 753$	< .001	$r_{rb} = 0.93$ [0.86 0.97]
Fear > disgust	t = -5.37	< .001	$d_z = -0.86$ [-1.22 -0.49]
Fear > surprise	t = -1.86	.07	$d_z = -0.3$ [-0.62 0.03]
Surprise > anger	$W = 771$	< .001	$r_{rb} = 0.98$ [0.95 0.99]
Surprise > disgust	t = -4.09	< .001	$d_z = -0.66$ [-1.0 -0.31]
Anger > disgust	$W = 0$	< .001	$r_{rb} = -1.0$

All df = 38. For each set of pairwise comparisons, minimum Bonferroni-Holm adjusted α = .0083.

S7 Table. Results of pairwise comparisons for the percentage fixation duration analyses of Experiment 2b: main effects of region of interest.

Fixation location contrast	*statistic*	*p*	effect size [95% CI]
	Angry faces		
Eyes > brow	$W = 780$	< .001	$r_{rb} = 1.0$
Eyes > nose	$t = 0.86$	.39	$d_z = 0.14$ [-0.18 0.45]
Eyes > mouth	$t = 7.92$	< .001	$d_z = 1.27$ [0.84 1.69]
Nose > brow	$W = 778$	< .001	$r_{rb} = 0.995$ [0.989 0.998]
Nose > mouth	$t = 7.17$	< .001	$d_z = 1.15$ [0.74 1.55]
Mouth > brow	$W = 458$	.35	$r_{rb} = 0.17$ [-0.18 0.49]
	Disgusted faces		
Eyes > brow	$t = 9.34$	< .001	$d_z = 1.5$ [1.03 1.95]
Eyes > nose	$t = -0.06$	.96	$d_z = -0.01$ [-0.32 0.31]
Eyes > mouth	$t = 3.96$	< .001	$d_z = 0.63$ [0.29 0.98]
Nose > brow	$t = 11.42$	< .001	$d_z = 1.83$ [1.31 2.34]
Nose > mouth	$t = 4.62$	< .001	$d_z = 0.74$ [0.38 1.09]
Mouth > brow	$t = 5.23$	< .001	$d_z = 0.84$ [0.47 1.2]
	Fearful faces		
Eyes > brow	$t = 12.58$	< .001	$d_z = 2.01$ [1.46 2.56]
Eyes > nose	$t = 2.35$	.024	$d_z = 0.38$ [0.05 0.7]
Eyes > mouth	$t = 7.3$	< .001	$d_z = 1.17$ [0.76 1.57]
Nose > brow	$W = 780$	< .001	$r_{rb} = 1.0$
Nose > mouth	$t = 4.43$	< .001	$d_z = 0.71$ [0.35 1.06]
Mouth > brow	$W = 743$	< .001	$r_{rb} = 0.91$ [0.81 0.95]
	Surprised faces		
Eyes > brow	$t = 13.52$	< .001	$d_z = 2.16$ [1.587 2.74]
Eyes > nose	$t = 2.83$	.007	$d_z = 0.45$ [0.12 0.78]
Eyes > mouth	$t = 7.31$	< .001	$d_z = 1.17$ [0.76 1.57]
Nose > brow	$W = 780$	< .001	$r_{rb} = 1.0$
Nose > mouth	$t = 4.28$	< .001	$d_z = 0.69$ [0.33 1.03]
Mouth > brow	$W = 775$	< .001	$r_{rb} = 0.99$ [0.97 1.0]

All df = 38. For each set of pairwise comparisons, minimum Bonferroni-Holm adjusted α = .0083.

Analysis of paths of first saccades after face onset in Experiment 2b

Do observers immediately saccade towards informative regions upon face onset, even when the face is presented for longer than in the brief-fixation experiments? To address this question, we conducted an exploratory analysis on the saccade path measures for valid first saccades from 38 participants. (We had not set out to test this question; it was suggested to us during the review process for this journal by one of the referees.) A 4 (emotion) × 6 (target location) repeated measures ANOVA was conducted to investigate whether initial saccades, operationalised as first saccades that happened after face onset, target emotion informative facial features. Contrary to the brief fixation paradigm, when participants made their initial saccades in the long presentation paradigm, they still had access to the whole image.

There were no significant main effects for emotion, $F(3, 104.1) = 1.47$, $p = .23$, $\eta p^2 = .04$, or target location, $F(1.71, 63.32) = 1.79$, $p = .18$, $\eta p^2 = .05$. There was a significant interaction between emotion and target location, $F(7, 36) = 16.94$, $p < .001$, $\eta p^2 = .31$. To further investigate the source of this interaction, separate one-way ANOVAs were carried out to test the effect of emotion for each target location.

For the central brow region, there was a main effect of emotion, $F(3,111) = 18.97$, $p < .001$, $\eta p^2 = .34$. Pairwise comparisons suggest that, as hypothesised, initial saccades towards the brow were stronger for angry expressions ($M = 0.551$, $SD = 0.201$) compared to all others (fear: $M = 0.514$, $SD = 0.197$, uncorrected $p = .006$, $d_z = 0.48$; surprise: $M = 0.512$, $SD = 0.208$, uncorrected $p < .001$, $d_z = 0.58$; disgust: $M = 0.46$, $SD = 0.205$, uncorrected $p < .001$, $d_z = 1.14$). Initial saccades towards the brow were least strong for disgust compared to all other emotions (all ps < .05).

There was a main effect of emotion for the mouth region, $F(3, 111) = 15.35$, $p < .001$, $\eta p^2 = .29$. Pairwise comparisons suggest that, as hypothesised, initial saccades more strongly targeted the mouth for surprised expressions ($M = 0.445$, $SD = 0.204$) compared to angry ($M = 0.39$, $SD = 0.176$, uncorrected $p < .001$, $d_z = 0.73$) and fearful ($M = 0.406$, $SD = 0.188$,

uncorrected $p < .001$, $d_z = 0.63$) expressions. Also as hypothesised, initial saccades were stronger towards the mouth for disgusted expressions (*M* = 0.464, *SD* = 0.198) compared to angry and fearful expressions (disgust ~ anger: $p < .001$, $d_z = 0.83$; disgust ~ fear: $p < .001$, $d_z = 0.79$).

For the left eye, there was a main effect of emotion, $F(3, 111) = 12.04$, $p < .001$, $\eta p^2 = .25$. Pairwise comparisons show that left eye was targeted least strongly for disgust expressions (*M* = 0.445, *SD* = 0.179) compared to all other emotions (anger: *M* = 0.496, *SD* = 0.174; fear: *M* = 0.495, *SD* = 0.183; surprise: *M* = 0.488, *SD* = 0.183; all uncorrected *p*s < .001, d_zs > 0.75).

For the right eye, there was a main effect of emotion, $F(3, 111) = 4.67$, $p = .004$, $\eta p^2 = .11$. Pairwise comparisons suggested that the right eye was targeted more strongly for angry expressions (*M* = 0.45, *SD* = 0.123) compared to fearful (*M* = 0.414, *SD* = 0.323, uncorrected $p < .001$, $d_z = 0.6$) and disgust (*M* = 0.42, *SD* = 0.121, uncorrected $p = .002$, $d_z = 0.54$) expressions only.

For the left cheek, there was also a main effect of emotion, $F(3, 111) = 3.02$, $p = .033$, $\eta p^2 = .08$. Pairwise comparisons showed no significant differences among emotions.

Finally, there was also a main effect of emotion for the right cheek, $F(3, 111) = 5.55$, $p = .001$, $\eta p^2 = .13$. Pairwise comparisons suggest that the right cheek was targeted more strongly for disgusted expressions (*M* = 0.475, *SD* = 0.124) compared to angry (*M* = 0.434, *SD* = 0.124, uncorrected $p = .001$, $d_z = 0.56$) and fearful (*M* = 0.433, *SD* = 0.128, uncorrected $p < .001$, $d_z = 0.67$) expressions.

Supplementary General Discussion

We found that, in the brief-fixation paradigms for both experiments, first saccades after image offset were directed upwards from initial fixation on the mouth more frequently than downwards from initial fixation on the eyes. Additionally, we found the same pattern for

saccades going upwards from the lower facial features (i.e., mouth and cheeks) compared to saccades going downwards from the upper face features (i.e., eyes and brow). These findings replicate previous findings of higher proportions of fixation changes upwards from the mouth than downwards from the eyes [4,8] or from the mid-point between the eyes [1–3,5–7]. These findings could be interpreted as reflecting a tendency for the reflexive saccades to be directed more strongly towards the eyes than towards the mouth, as other researchers have tended to do [e.g., 3,4,7], yet our saccade path analyses do not support such an interpretation (as reported and discussed in the main article).

We also found that the propensity to saccade upwards or downwards was modulated by the expression presented but this was not constant across the two experiments. Contrary to the findings of Gamer and colleagues, we did not find an effect of expression on the proportion of saccades going upwards from the lower-face features. It is possible that observers in our experiments (especially in Experiment 2a) found the mouth region more informative and therefore did not make as many saccades upwards from the mouth as in the previous studies. By contrast, expression did have an effect on the proportion of saccades going downwards from the upper-face features; specifically, there were fewer saccades going downwards from the eyes for sad faces for Experiment 1 and there were more saccades going downwards from the eyes for fear and surprised faces compared to angry faces in Experiment 2a. We suggest that this reflects the relative informativeness of the mouth for fearful and surprised expressions compared to the brow for angry expressions.

When looking at the end location of reflexive saccades, we show that these saccades end most frequently in the nose region regardless of expression. This can be an indication of center-of-gravity effect as suggested by Bindemann et al. [9] where first saccades, especially those within the first 250ms of face onset, target the centre of the face. However, it has to be noted that the nose region, which is used as an index for the centre of face in this analysis,

covers the region of the face from the bridge of the nose to the apex of the nose. Therefore, the saccades ending within the nose region might also be targeting the region just below the eyes. This region was suggested by Hsiao and Cottrell [11] and Peterson and Eckstein [12] to be the optimal viewing position for face recognition.

The results of our proportion saccade analyses suggest that observers make reflexive saccades upwards from the mouth of expressive faces more often than they do downwards from the eyes; however, our results do not allow us to argue that these saccades are consistently targeting informative features. One possible reason for the difference between our studies and the previous ones is their use of a more limited range of expressions, namely fear, happy and neutral (and in some cases, also anger). The informative regions for happy and fearful faces are spread out in different sections of the face [13,14]. While the most informative region for happy faces is the mouth, the most informative region for fearful faces is the eye region [13–15]. It is possible that this separation of informative features between these two expressions might have accentuated the difference in reflexive saccade patterns. Additionally, since the information in the eye region of fearful and surprised faces is similar, our participants might not have found the eye region more useful to identify fearful expressions than to identify any other expression leading to no clear difference in reflexive saccade patterns from the mouth region upwards.

Although the results of the saccade path analyses for the brief-fixation experiments do not provide support for the hypothesis that first saccades target emotion-informative facial features, an equivalent (though exploratory) analysis of the saccade path measures for first saccades after face onset in Experiment 2b, in which the faces were presented for longer, did in fact provide evidence consistent with that hypothesis. Specifically, first saccades were more strongly in the direction of the central brow for angry compared to fearful, surprised

and disgusted faces, and more strongly towards the mouth for surprised and disgusted faces than for angry or fearful faces.

We found that the latencies of the first reflexive saccades were affected by the initial fixation location on the face image for both Experiments 1 and 2a. Observers made quicker saccades from the initial fixation locations at the cheeks compared to all other initial fixation locations. It has been shown that latency to saccade to an emotional face (fearful and happy) presented briefly in the periphery is shorter compared to the latency to saccade to a neutral face suggesting that saccade programming is affected by the emotional content of the face [16]. It has also been suggested that saccade latency can be a reliable index of spatial attention orientation [17]. These researchers showed that saccade latencies to a cued location are shorter compared to a non-cued location. It is possible, therefore, that in our studies the emotional content in the rest of the face acted as a cue for the selection of the next saccade target leading our participants to make a quicker saccade towards another, more expression-informative facial feature. Similarly, Arizpe et al. [18] showed that the saccade latencies from a central location on the face were longer compared to peripheral fixation locations that did not correspond to any of the internal facial features. The central location on a face is suggested to contain most visual information making this the optimal fixation location for face-related tasks [11,12,19]. Additionally, the saccade generation model by Findlay and Walker [10] suggests that the trigger to move from a fixated position depends on the amount of cognitive and perceptual processing the currently fixated location requires. Since the initial fixation locations in both of our experiments consisted of internal features that are considered informative (i.e., the eyes, the brow and the mouth), it is possible that there was more information to be processed at initial fixation locations other than the cheeks, leading our observers to saccade away from these features much slower compared to the cheeks.

Supplementary References

1. Boll S, Gamer M. 5-HTTLPR modulates the recognition accuracy and exploration of emotional facial expressions. Frontiers in Behavioral Neuroscience. 2014;8. doi:10.3389/fnbeh.2014.00255

2. Gamer M, Zurowski B, Büchel C. Different amygdala subregions mediate valence-related and attentional effects of oxytocin in humans. Proceedings of the National Academy of Sciences. 2010;107: 9400–9405. doi:10.1073/pnas.1000985107

3. Gamer M, Schmitz AK, Tittgemeyer M, Schilbach L. The human amygdala drives reflexive orienting towards facial features. Current Biology. 2013;23: R917–R918. doi:http://dx.doi.org/10.1016/j.cub.2013.09.008

4. Gamer M, Büchel C. Amygdala activation predicts gaze toward fearful eyes. J Neurosci. 2009;29: 9123–9126. doi:10.1523/jneurosci.1883-09.2009

5. Kliemann D, Dziobek I, Hatri A, Steimke R, Heekeren HR. Atypical reflexive gaze patterns on emotional faces in autism spectrum disorders. The Journal of Neuroscience. 2010;30: 12281–12287. doi:10.1523/jneurosci.0688-10.2010

6. Kliemann D, Dziobek I, Hatri A, Baudewig J, Heekeren HR. The role of the amygdala in atypical gaze on emotional faces in Autism Spectrum Disorders. The Journal of Neuroscience. 2012;32: 9469–9476. doi:10.1523/jneurosci.5294-11.2012

7. Scheller E, Büchel C, Gamer M. Diagnostic features of emotional expressions are processed preferentially. PLoS ONE. 2012;7: e41792. doi:10.1371/journal.pone.0041792

8. Atkinson AP, Smithson HE. The impact on emotion classification performance and gaze behavior of foveal versus extrafoveal processing of facial features. Journal of Experimental Psychology: Human Perception & Performance. 2020;46: 292–312. doi:10.1037/xhp0000712

9. Bindemann M, Scheepers C, Burton AM. Viewpoint and center of gravity affect eye movements to human faces. Journal of Vision. 2009;9: 1–16. doi:10.1167/9.2.7

10. Findlay JM, Walker R. A model of saccade generation based on parallel processing and competitive inhibition. Behavioral and Brain Sciences. 1999;22: 661–674. doi:10.1017/S0140525X99002150

11. Hsiao JH, Cottrell GW. Two fixations suffice in face recognition. Psychological Science. 2008;19: 998–1006. doi:10.1111/j.1467-9280.2008.02191.x

12. Peterson MF, Eckstein MP. Looking just below the eyes is optimal across face recognition tasks. Proceedings of the National Academy of Sciences. 2012;109: E3314–E3323. doi:10.1073/pnas.1214269109

13. Beaudry O, Roy-Charland A, Perron M, Cormier I, Tapp R. Featural processing in recognition of emotional facial expressions. Cognition & Emotion. 2014;28: 416–432. doi:10.1080/02699931.2013.833500

14. Calder AJ, Young AW, Keane J, Dean M. Configural information in facial expression perception. J Exp Psychol Hum Percept Perform. 2000;26: 527–551. doi:10.I037/TO96-1523.26.2.527

15. Smith ML, Cottrell GW, Gosselin F, Schyns PG. Transmitting and decoding facial expressions. Psychological Science. 2005;16: 184–189. doi:10.1111/j.0956-7976.2005.00801.x

16. Bannerman RL, Hibbard PB, Chalmers K, Sahraie A. Saccadic latency is modulated by emotional content of spatially filtered face stimuli. Emotion. 2012;12: 1384–1392. doi:10.1037/a0028677

17. Şentürk G, Greenberg AS, Liu T. Saccade latency indexes exogenous and endogenous object-based attention. Attention, Perception, and Psychophysics. 2016;78: 1998–2013. doi:10.3758/s13414-016-1136-1

18. Arizpe J, Kravitz DJ, Yovel G, Baker CI. Start position strongly influences fixation patterns during face processing: Difficulties with eye movements as a measure of information use. PLoS ONE. 2012;7. doi:10.1371/journal.pone.0031106

19. Or CCF, Peterson MF, Eckstein MP. Initial eye movements during face identification are optimal and similar across cultures. Journal of Vision. 2015;15: 12–12. doi:10.1167/15.13.12
